# Biogeography of Southern Ocean prokaryotes: a comparison of the Indian and Pacific sectors

**DOI:** 10.1111/1462-2920.15906

**Published:** 2022-02-16

**Authors:** Swan L. S. Sow, Mark V. Brown, Laurence J. Clarke, Andrew Bissett, Jodie van de Kamp, Thomas W. Trull, Eric J. Raes, Justin R. Seymour, Anna R. Bramucci, Martin Ostrowski, Philip W. Boyd, Bruce E. Deagle, Paula C. Pardo, Bernadette M. Sloyan, Levente Bodrossy

**Affiliations:** ^1^ Institute for Marine and Antarctic Studies, University of Tasmania Hobart TAS 7000 Australia; ^2^ Oceans and Atmosphere, Commonwealth Scientific and Industrial Research Organisation Hobart TAS 7000 Australia; ^3^ School of Environmental and Life Sciences, University of Newcastle Callaghan NSW 2308 Australia; ^4^ Australian Antarctic Division, Channel Highway Kingston TAS 7050 Australia; ^5^ Antarctic Climate and Ecosystem Cooperative Research Center (ACE‐CRC) Hobart TAS 7000 Australia; ^6^ Flourishing Oceans, Minderoo Foundation, Broadway Nedlands WA 6009 Australia; ^7^ Climate Change Cluster, University of Technology Sydney Ultimo NSW 2007 Australia; ^8^ National Collections & Marine Infrastructure, Commonwealth Scientific and Industrial Research Organisation Hobart TAS 7000 Australia; ^9^ Departamento de Oceanografía, Instituto de Investigacións Mariñas (IIM‐CSIC) Vigo Pontevedra 36208 Spain

## Abstract

We investigated the Southern Ocean (SO) prokaryote community structure via zero‐radius operational taxonomic unit (zOTU) libraries generated from 16S rRNA gene sequencing of 223 full water column profiles. Samples reveal the prokaryote diversity trend between discrete water masses across multiple depths and latitudes in Indian (71–99°E, summer) and Pacific (170–174°W, autumn‐winter) sectors of the SO. At higher taxonomic levels (phylum‐family) we observed water masses to harbour distinct communities across both sectors, but observed sectorial variations at lower taxonomic levels (genus‐zOTU) and relative abundance shifts for key taxa such as Flavobacteria, SAR324/Marinimicrobia, *Nitrosopumilus* and *Nitrosopelagicus* at both epi‐ and bathy‐abyssopelagic water masses. Common surface bacteria were abundant in several deep‐water masses and vice‐versa suggesting connectivity between surface and deep‐water microbial assemblages. Bacteria from same‐sector Antarctic Bottom Water samples showed patchy, high beta‐diversity which did not correlate well with measured environmental parameters or geographical distance. Unconventional depth distribution patterns were observed for key archaeal groups: Crenarchaeota was found across all depths in the water column and persistent high relative abundances of common epipelagic archaeon *Nitrosopelagicus* was observed in deep‐water masses. Our findings reveal substantial regional variability of SO prokaryote assemblages that we argue should be considered in wide‐scale SO ecosystem microbial modelling.

## Introduction

Despite being persistently cold and having little to no sunlight in the winter, considerable areas of the Southern Ocean (SO) are biologically productive regions that play central roles in nutrient recirculation, supporting primary production across global ocean scales and mediating CO_2_ exchange and sequestration via the biological pump (Álvarez *et al*., 2002; Arrigo *et al*., 2008; Lenton *et al*., 2013; Legge *et al*., 2015). The SO is also an important region for recirculation of climate active trace gasses such as dimethyl sulfoxide and methane (Curran and Jones, 2000; Gabric *et al*., 2001; Wadham *et al*., 2012; Thurber *et al*., 2020). Prokaryotes (domain *Bacteria* and *Archaea*) make up the largest proportion of microbial biomass in open SO waters and are primary drivers of biogeochemical cycling (Wilkins *et al*., 2013b). Even with the recent global ocean microbial surveys (Rusch *et al*., 2007; Duarte, 2015; Sunagawa *et al*., 2015; Salazar *et al*., 2016), the diversity of SO prokaryotes remains understudied, with previous studies performed mostly within single locations or specific oceanic regions (Selje *et al*., 2004; Murray and Grzymski, 2007; Alonso‐Saez *et al*., 2011; Grzymski *et al*., 2012; Wilkins *et al*., 2013a; Wilkins *et al*., 2013c; Signori *et al*., 2014; Yu *et al*., 2015; Landa *et al*., 2016; Milici *et al*., 2017; Liu *et al*., 2019; Liu *et al*., 2020). Because of the fundamental ecosystem services provided by SO prokaryotes, the development of an accurate baseline understanding of the key players and their biogeographical controls at broad, multi‐sector scales is essential for better predicting the consequences of global climate change on the SO ecosystem function and biogeochemistry. Furthermore, a general lack of knowledge of the SO prokaryotic diversity in the aphotic zone, which is estimated to globally contain up to 75% of prokaryotic biomass and production respectively, also limits our understanding of microbial functions within this critical dark end of the biological pump (Arístegui *et al*., 2009; Herndl and Reinthaler, 2013; Shah Walter *et al*., 2018).

Due to their small size, spatial ubiquity, relatively short community turnover time, constant passive movement and recirculation, identifying the distribution patterns of marine microbes, and disentangling the potential environmental (physical and chemical) and biological factors that drive these observed patterns have been a persistent challenge in marine microbial ecology (Tittensor *et al*., 2010; Beaugrand and Kirby, 2018; Raes *et al*., 2018). The prokaryotes in the SO are no exception. Traditionally, depth, latitude, season, spatial distance and water properties were thought as key contributors to influencing the distribution of marine prokaryotes (Gilbert *et al*., 2012; Zinger *et al*., 2014; Fuhrman *et al*., 2015; Parada and Fuhrman, 2017), but more recently advection, historical contingency, primary productivity and trophic interactions are also thought to play a role (Wilkins *et al*., 2013a; Fukami, 2015; Raes *et al*., 2018; Gralka *et al*., 2020).

The water properties temperature, salinity, oxygen and nutrients that are primarily used to oceanographically define water masses (i.e. water bodies with similar water properties) (Gordon, 1967; Emery, 2003) have been thought to act as boundaries delineating microbial distribution (Richardson and Schoeman, 2004; Brown *et al*., 2012; Wilkins *et al*., 2013c; Baltar and Arístegui, 2017) and past research has shown microbial communities to be distinct between water masses (Galand *et al*., 2010; Agogue *et al*., 2011; Wilkins *et al*., 2013a; Salazar *et al*., 2016; Zoccarato *et al*., 2016). Deep SO water masses, incorporating mainly the circumpolar deep waters and Antarctic Bottom waters (AABW) are spatially widespread. They are oceanographically categorized as common water mass types across a wide range of latitudes and sectors (i.e. different longitudinal regions, e.g. Indian, Atlantic and Pacific sectors of the SO) since they are considered uniform environments based on the water properties they are defined by. This may initially suggest the deep ocean support lower abundances and diversity of prokaryotes, however growing number of studies show high levels of diversity in deep water masses (Hewson *et al*., 2006; Arístegui *et al*., 2009; Nagata *et al*., 2010). This high diversity has been attributed to reduced connectivity and microbial dispersal due to sluggish deep sea currents, the presence of deep sea ridges, variable primary productivity and flux of particles from the surface, as well as water mass age (Varela *et al*., 2008; Schauer *et al*., 2010; Zinger *et al*., 2011; Salazar *et al*., 2016; Mestre *et al*., 2018). Much remains unknown about the community composition and drivers of observed microbial assemblage within widespread water masses that are separated across large spatial ranges of thousands of kilometres, such as within the deep SO water masses in different oceanic sectors.

Here, we explore the SO prokaryote community composition using 16S rRNA metabarcoding data associated with 223 water samples that were processed as high‐resolution, single nucleotide varying zero‐radius operational taxonomic units (zOTUs). Samples were collected from 3 to 8 depths spanning the full water column of 58 stations, representing all major water masses across the Indian and Pacific sectors of the SO during the austral summer and winter. The sequence dataset, together with distance‐based linear modelling (DistLM) with the corresponding environmental variables and distance–decay analysis allowed us to take a deep dive into (i) whether and how much do prokaryote communities in each of the hydrographically defined epi‐, meso‐ and abyssopelagic water masses differ across different SO sectors that have high spatial separation. We explored which taxonomic groups and at which taxonomic level the community variation(s) were observed, and (ii) compared deep water mass samples across oceanic sectors at an expanded scope. This is something that is not yet prevalent within the SO and provides scientific evidence of the varying community composition in oceanographically similar deep water masses in addition to exploring vertical connectivity of microbial distribution across large spatial distances. Finally, we explored (iii) which factors (such as depth, hydrographic variables and/or geographic distance) were the dominant drivers of the observed prokaryote community composition patterns.

## Results and discussion

**Fig. 1:**
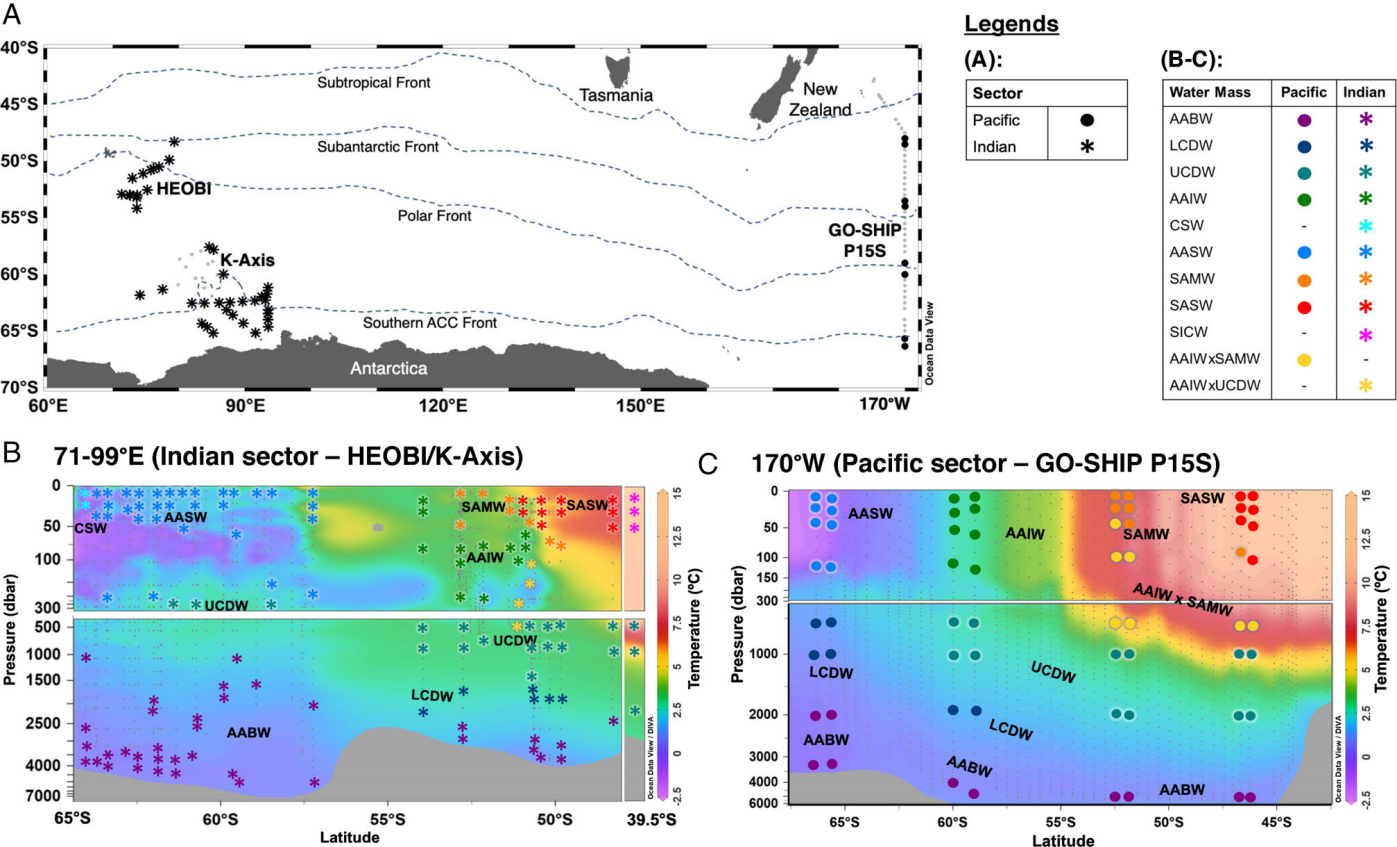
A. Map of microbial sampling stations within the HEOBI, K‐Axis and GO‐SHIP P15S transects. Stations where full‐depth microbial profile analysis were conducted are indicated based on the longitudinal sector they belong to by a closed circle (Pacific sector samples) or bold star (Indian sector samples). Frontal locations indicated are approximate latitudes only. Cross‐section showing vertical distribution of temperature at B. 71–99°E (HEOBI and K‐Axis transects) and C. 170°W (P15S transect), as well as microbial sampling locations and their distribution (indicated by coloured symbols based on the water mass they are categorized to) across the major water masses. Smaller grey dots indicate sampling points for the temperature profile. AABW – Antarctic Bottom Water; LCDW – Lower Circumpolar Deep Water; UCDW – Upper Circumpolar Deep Water; AAIW – Antarctic Intermediate Water; CSW – Continental Shelf Water; AASW – Antarctic Surface Water; SAMW – Subantarctic Mode Water; Subantarctic Surface Water; SICW – South Indian Central Water. Several samples were identified to harbour temperature‐salinity characteristics of two different water masses (regions where water mass mixing occurred): AAIWxSAWM – water with mixed characteristics of AAIW and SAMW; AAIWxUCDW – water with mixed characteristics of AAIW and UCDW.

This study compared data from the Pacific and Indian sectors of the SO. Cruises were conducted during late autumn‐early winter for the Pacific sector and summer for the Indian sector. Sampling encompassed the surface to deep (bathypelagic) water spectrum and covered eight water masses within the Pacific and 10 within the Indian sector (Fig. 1), where the water masses were defined by temperature‐salinity and oxygen distribution plots (Figs. S1 and S2). Information (oceanographic voyage, sector, water mass) and number of samples collected per location are specified in the Experimental procedures section. Detailed water property profiles, definitions of SO water masses and fronts within this study are provided in supporting information. Notably, surface water salinity (32.7–33.9; PSS‐78) at 60–65°S within the Indian sector was observed to be lower than usual due to sea ice meltwater originating from the Antarctic Shelf Current (Bestley *et al*., 2020). Deep waters within the Pacific sector also had lower dissolved oxygen (DO) levels (252–299 μmol L^−1^) compared to the Indian sector (320–354 μmol L^−1^) (Table S1).

After QC, our dataset yielded 17.5 and 14.6 million bacterial and archaeal sequencing reads respectively, and following subsampling, resulted in a total of 30 209 bacterial and 10 048 archaeal zOTUs. The alpha‐diversity indices (Table S2) and rarefaction curves (Fig. S3; generated per sample for both bacteria and archaea) indicated microbial richness to be comprehensively represented, with Good's coverage of >0.92 demonstrating that at least 92% of the total species per sample were being considered in the study (Fig. S3; Table S2). Species richness was highest in mixed water masses (i.e. AAIWxSAMW – see Fig. 1 legend and Experimental procedures for all water mass definitions) and lowest in colder AASW and CSW surface water masses for both bacteria and archaea, corroborated by Chao1 true species richness approximations (Table S2; Fig. S4). The increased species diversity observed at the mixed water masses is most likely associated with increased resource availability and higher primary productivity known to occur at frontal zones and areas of water mass transitions where there is elevated mixing and turbulence [Scales *et al*., 2014 and references therein].

### Depth, water mass and sector define unique prokaryote communities

**Fig. 2:**
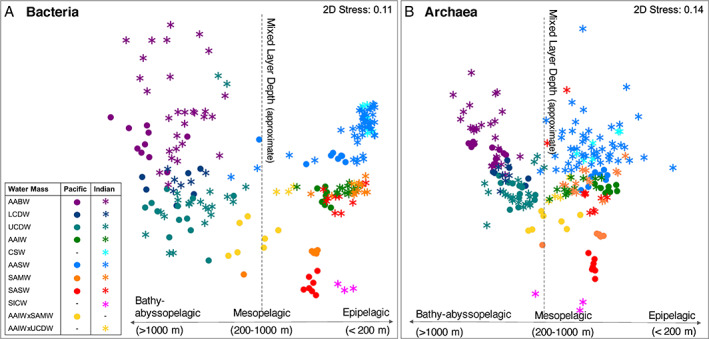
nMDS plots of (A) bacterial and (B) archaeal 16S rRNA gene communities distribution patterns generated based on zOTU abundance tables. Water mass name acronyms are as indicated in Fig. 1.

**Fig. 3:**
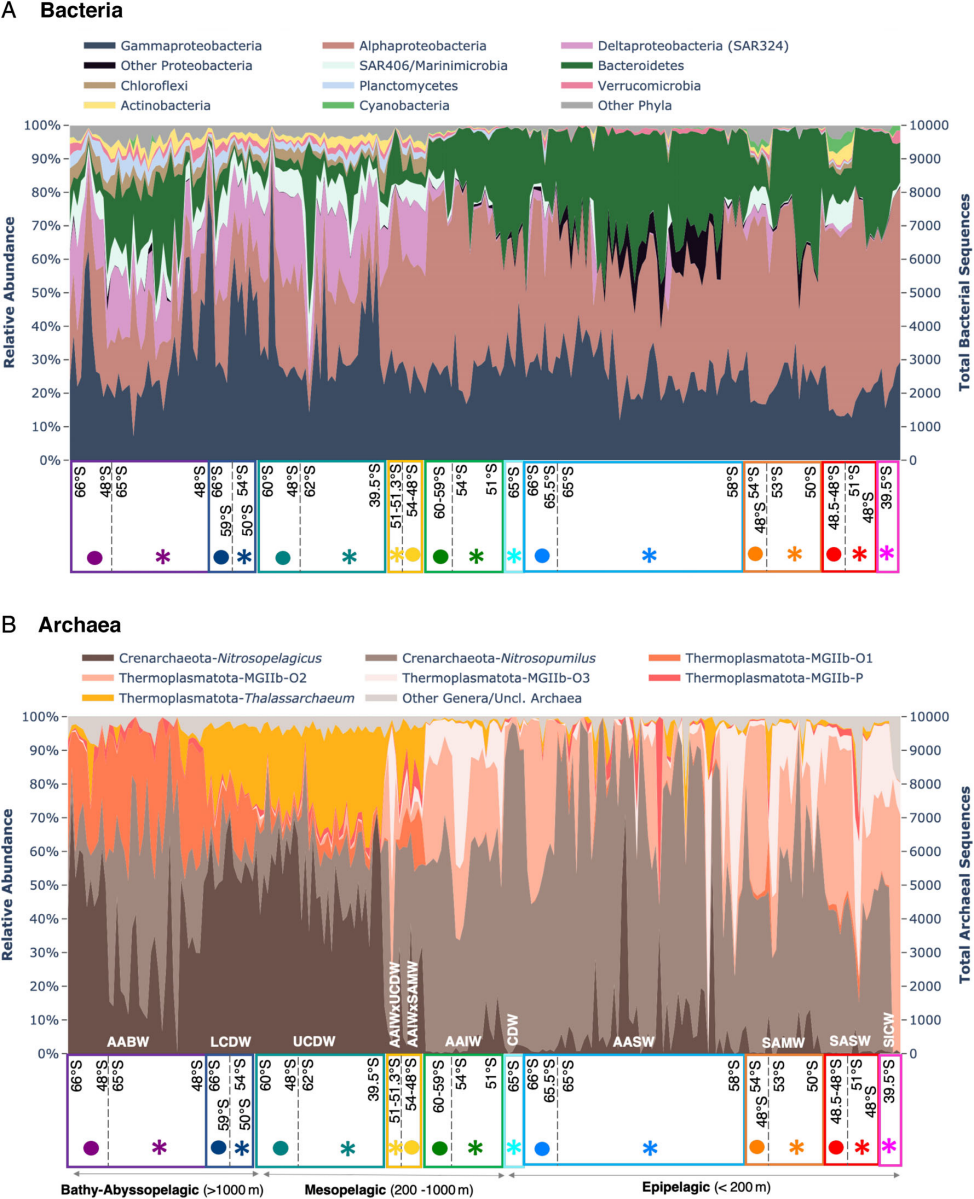
Relative abundance of major (A) bacteria and (B) archaea detected from the different water masses at phylum/class level. ‘Other phyla’ or ‘Other genera’ are bacterial phylum or archaeal genera with overall mean relative abundances of <0.5% across all water masses. ‘Unclassified archaea’ include archaeal zOTUs that were classified with bootstrap confidence values <50%. For each water mass type, samples are arranged by decreasing latitude from left to right, first for the Pacific and followed by Indian sector samples. Water column depth categories: Bathy‐Abyssopelagic (>1000 m), Mesopelagic (200–1000 m), and Epipelagic (<200 m) provide a rough depth indication of the different water masses. Full charts showing each of the 223 samples on the *x*‐axis are in Fig. S6. Water mass name acronyms are as indicated in Figs. 1 and 2 legends.

**Fig. 4:**
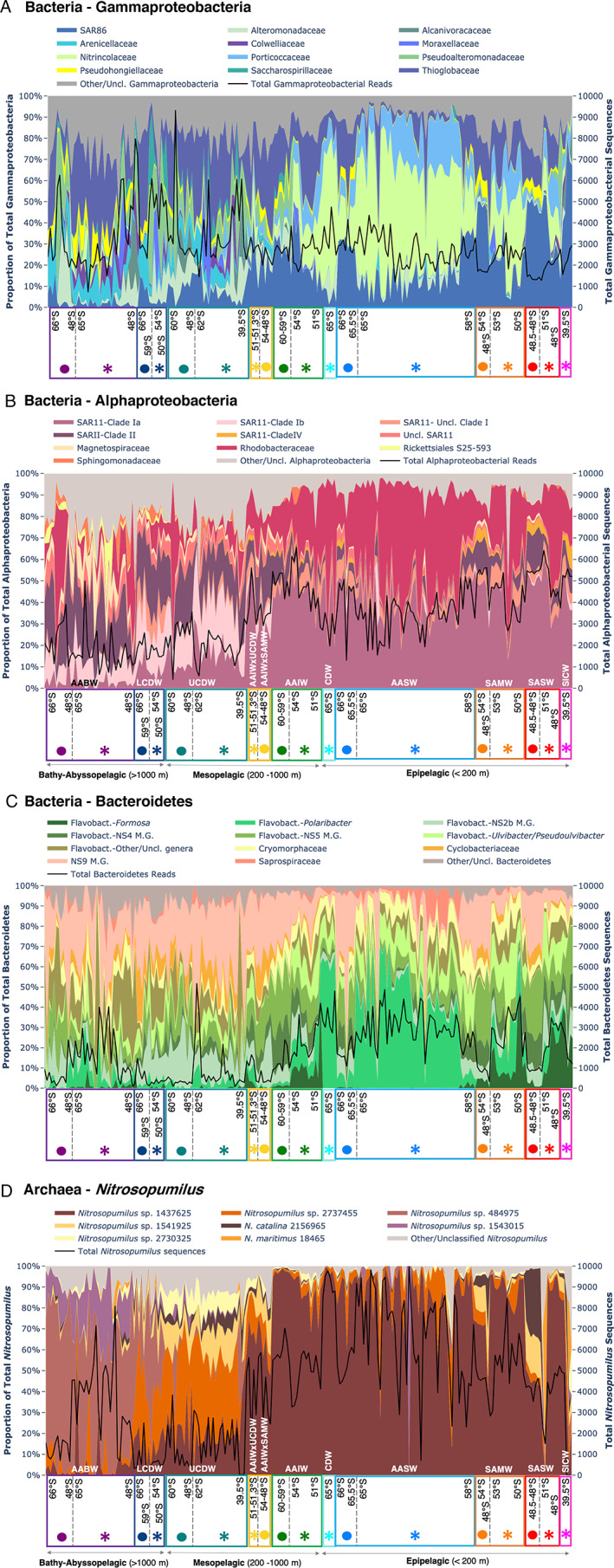
Detailed charts of the main families within (A) Gammaproteobacteria, (B) Alphaproteobacteria and (C) Bacteroidetes. Relative abundances of main species/zOTUs within the archaeal *Nitrosopumilus* genus are shown in (D). Samples are arranged from high to low latitude in each water mass for the Pacific and Indian sector as indicated by their respective sector and water mass symbols (see Fig. 1/Fig. 2 legends). M.G. – Marine Group.

**Fig. 5:**
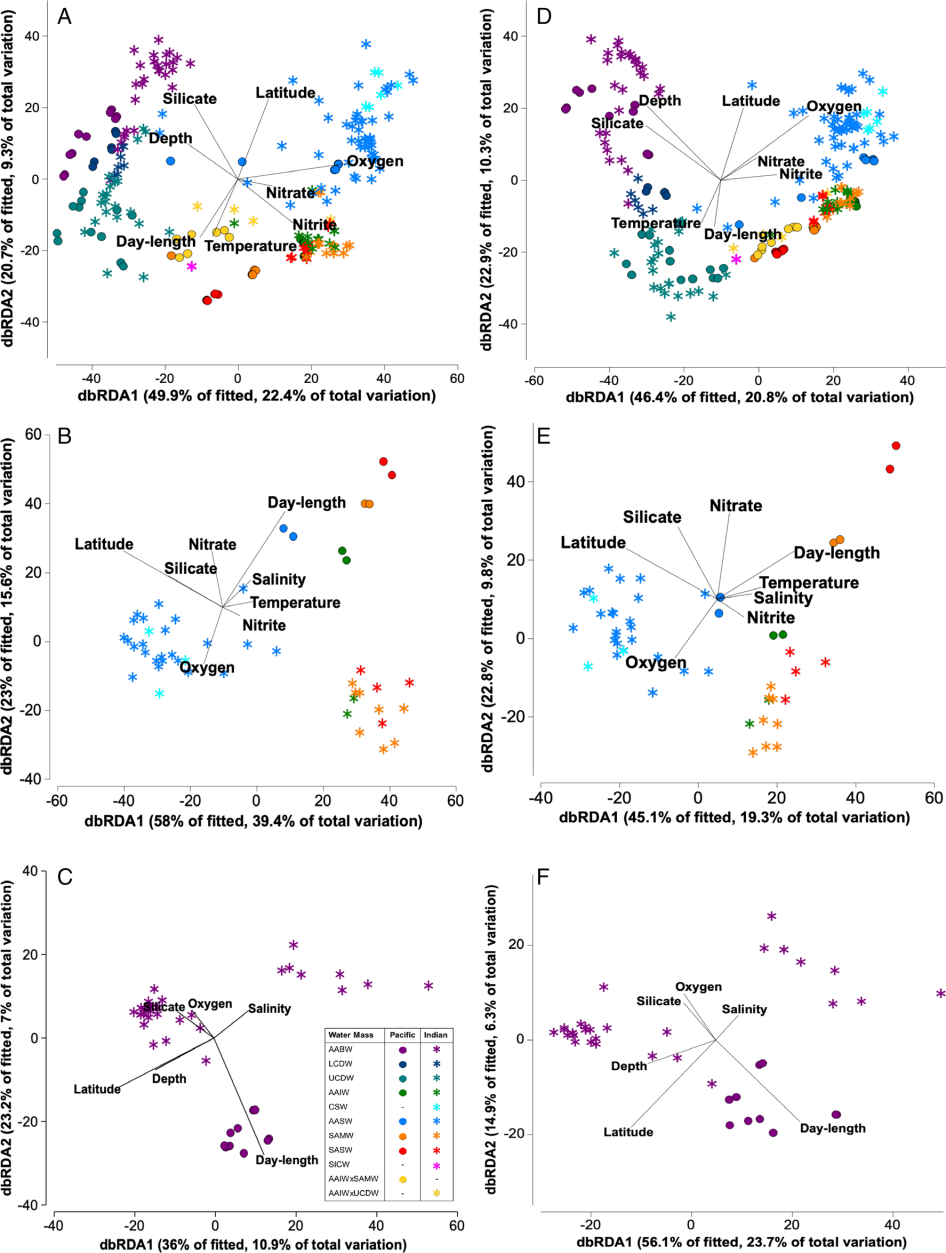
Distance‐based redundancy analysis (dbRDA) ordination of fitted values from DistLM models of the correlation between bacterial (A–C) and archaeal (D–F) community composition with environmental and geographical variables. Model ordinations of bacterial and archaeal communities from all water masses (A, D) as well as community subsets from surface water samples (B, E) and AABW (C, F) are shown. The effect of each environmental/geographical (predictor) variable is represented by vectors on two visualized axes explaining the most community variation. Relative strength of the correlation between the predictor variable with the dbRDA axes corresponds to vector length, while correlation of the predictor variable to the visualized axes corresponds to vector direction. Surface samples analyzed include all samples taken from depths <20 m from all stations. Water mass name acronyms are as indicated in Fig. 1.

As found in previous studies (Field *et al*., 1997; López‐García *et al*., 2001; DeLong *et al*., 2006; Brown *et al*., 2009; Fu *et al*., 2013), the bacterial and archaeal communities were first, strongly structured by depth; large composition differences between communities above and below the mixed layer depth (MLD) were observed through their beta‐diversity visualized in the nMDS (Fig. 2). This observation was supported by significant (*P*_bacteria_ and *P*_archaeea_ ≤ 0.0001) permutational multivariate ANOVA (PERMANOVA) tests (Table S3A) where the heterogeneity was not likely to be an effect of multivariate dispersion (Tests of homogeneity of dispersions [PERMDISP] – bacteria: *F*_1,221_ = 9.0934, *p* = 0.0053; archaea: *F*_1,220_ = 4.2361, *p* = 0.0548) (Table S3A). Relative abundance plots of the bacterial and archaeal community composition (Fig. 3) further detailed this community composition difference; the communities below the MLD displayed higher relative abundances of several clades, namely, Deltaproteobacteria, SAR406/Marinimicrobia, Chloroflexi, Planctomycetes, Actinobacteria for the bacterial community (Fig. 3A) and *Nitrosopelagicus*, Thermoplasmatota MGIIb‐O1 and MGIIb‐O1 for the archaea (Fig. 3B). Conversely, higher relative abundances of Alphaproteobacteria (SAR11 Clade Ia, Rhodobacteraceae), Bacteroidetes (Flavobacteria), *Nitrosopumilus* and Thermoplasmatota MGIIb‐O2 were observed in communities above the MLD (Figs. 3 and 4B and C). We also found DO (a co‐variable of depth) to correlate most strongly with bacterial and archaeal alpha diversity (Fig. S5), which further reinforces the depth‐related structuring of the prokaryote community in this study along with the DistLM analyses (Table S5) and dbRDA ordinations (Fig. 5A and D) which were used to assess the environmental and geographical predictors that best explained community distributions. The first axis of dBRDA ordinations corresponded with the strong separation of communities above and below the MLD observed in the nMDS (Fig. 2) and reflected large disparities between their oxygen levels (dbRDA1; bacteria *r*_oxygen_ = 0.71, archaeal *r*_oxygen_ = 0.57; Fig. S1, S2) These analyses demonstrated oxygen and depth significantly (*p* = 0.001) explain the largest and second‐largest proportion of variation in both the bacterial (DistLM: oxygen – 22%; depth – 13%) and archaeal (DistLM: oxygen – 19%; depth – 17%) communities (Table S5).

Persistent oceanographic features such as water masses can act as ecological boundaries, limiting prokaryote dispersal and/or successful colonization, and thus are known to structure microbial communities (Abell and Bowman, 2005b; Giebel *et al*., 2009; Galand *et al*., 2010; Zinger *et al*., 2011), but the effects of sector on structuring microbial communities within the same water mass are poorly investigated. Following depth, the prokaryote community in this study was also strongly structured by water mass and oceanic sector (hereafter referred to simply as ‘sector’), as shown in the nMDS plots and PERMANOVA tests (Fig. 2; Table S3B). PERMANOVA indicated sector to structure 9 the prokaryotes more strongly above the MLD while the effects of water mass characteristics explained a greater proportion of the prokaryote community variation below the MLD (Table S3B; *p* ≤ 0.0001 for all tests). There was evidence for interaction between water mass and sector effects in the epipelagic community (*p* ≤ 0.001), though effects from the interaction of the two factors explained smaller proportions of community variation compared to either sector or water masses alone (Table S3B). The effects of water mass and water mass × sector on the archaeal community above the MLD and the effects of sector and water mass × sector on the archaeal community below the MLD may have also been influenced by heterogeneity in multivariate dispersion (Table S3C).

### Dominant bacterial and archaeal clades in the different water masses and sectors

The prevailing bacterial and archaeal clades at higher taxonomic levels in the various water masses above and below the MLD are illustrated in Fig. 3 and as briefly summarized in the previous section. We further explored the dominant (high relative abundance/sequence numbers) bacteria (Alphaproteobacteria, Gammaproteobacteria, Bacteroidetes) and archaea (*Nitrosopumilus*) at finer taxonomic levels (Fig. 5). In brief, some notable distribution patterns include those from the SAR11 Clade 1a (13%–61% of Alphaproteobacteria). Out of 246 474 949 SAR11 sequences from all samples, 36.3% belonged to a single Clade 1a SAR11 zOTU, which displayed 100% similarity over the analyzed 435 bp V1–V3 region with Candidatus Pelagibacter ubique isolate HTCC1062. SAR11 Clade 1a, known to be dominant only in epipelagic waters (Giovannoni, 2017), was also observed to be still relatively abundant (10%–20%) down to 2000 m (and Fig. 5B). Rhodobacteraceae (3%–74% of Alphaproteobacteria) were dominated mainly by the genus *Planktomarina* and *Loktanella*. *Planktomarina*, belonging to the widely distributed RCA cluster was previously isolated from the North Sea where it is abundant but have also been found in the West Antarctic Peninsula (WAP) and central Kerguelen Plateau (Giebel *et al*., 2011; Alcaman‐Arias *et al*., 2018; Liu *et al*., 2020).

Overall, the archaeal community comprised primarily of the phylum Crenarchaeota (formerly Thaumarchaeota) and Thermoplasmatota (formerly Marine Group II, referred to as MGII in this article) with a slightly more prevalent presence of Crenarchaeota across water masses and sectors, except for SICW where the archaeal community comprised almost completely of MGII (Fig. 3B). The effects of sector in structuring community composition were observed and discussed in the following sections.

### Community composition of surface waters vary between the Indian and Pacific sectors

Sector was a key factor structuring the prokaryote community above the MLD (Table S3B), and its effects were observed through zonal geographical variations in dominant bacterial and archaeal taxa. As observed from Figs. 3 and 5, samples from the same water masses and similar depths harboured different communities at the Indian versus Pacific sectors even when observed at various taxonomic levels. Notably, Flavobacteria, a prevailing Bacteroidetes throughout this dataset and key player in phytoplankton‐derived organic matter degradation (Kirchman, 2002), was dominated by *Polaribacter* in the Indian AASW, but by the NS4, NS5 and NS2b groups in the Pacific AASW (Fig. 4C, Fig. S6d). With the Gammaproteobacteria, higher relative abundances of Thioglobaceae were observed within the Pacific while Porticoccaceae and Nitrincolaceae families were more abundant in the Indian sector (Fig. 4C, Fig. S6b, Fig. S7C). SAR324 and Marinimicrobia, clades known to be more common in the deeper waters with roles in carbon fixation, sulfur cycling and nitrous oxide reduction (Swan *et al*., 2011; Wright *et al*., 2014; Hawley *et al*., 2017), were only abundant in epipelagic SAMW‐SASW of the Pacific sector (SAR324: 1.2%–15%; Marinimicrobia: 6.1%–8.8%; Fig. 3A, Fig. S6a, Fig. S7A). For the archaea, species‐level variations of the dominant *Nitrosopumilus* genus were observed between sectors in the SAMW‐SASW (15%–85% of all archaea; Fig. 4D, Fig. S6f, Fig. S7F), with substantial percentages of *Nitrosopumilus catalina* (4%–36% of all *Nitrosopumilus*) and *Nitrosopumilus* sp. 1541925 found only in Pacific sector samples compared to Indian SAMW‐SASW which comprised almost entirely of *Nitrosopumilus* sp. 1437525 (Fig. 4D, Fig. S7F). The sectorial variation of these taxa was also corroborated by a substantial contribution to overall community dissimilarity between the water mass they were sampled from in the Pacific sector with the same corresponding water mass in the Indian sector (pair‐wise grouped SIMPER analysis, Table S4).

When we correlated environmental and geographical predictors to the surface prokaryote community distributions with DistLM and dBRDA plots, both types of predictors combined explained substantial proportions of prokaryote compositional variation (DistLM – bacteria: 68%, archaea: 42%; Table S9). Significant (*p* = 0.0001) proportions of the community variation were explained by latitude (bacteria – 32%, archaea – 16%), oxygen (bacteria – 26%, archaea – 16%) and day‐length (bacteria – 25%, archaea – 13%) (Table S5). We noted that strong correlations of the prokaryote community composition to day‐length were observed on the second dBRDA axis (Fig. 5B and E), which also showed the separation of Pacific and Indian sector samples that reflected the differences in the dominant taxa observed between sectors (i.e. Flavobacteria, *N*. *catalina* and several others as detailed in Figs. 3 and 4) within the same water masses (AASW, SAMW, SASW). While this does not conclusively disentangle the effects of sector (ocean circulation/distance) and seasonality, the strong correlation of day‐length, a strong proxy for seasonal variations (Gilbert *et al*., 2010) to the surface prokaryote community variations suggests seasonality as a plausible driver contributing to the observed sector specificity. Seasonality has been shown to widely influence microbial diversity patterns of some taxa in various ocean basins (Treusch *et al*., 2009; Gilbert *et al*., 2012; Giovannoni and Vergin, 2012; Cram *et al*., 2015; Signori *et al*., 2017) and the effects of seasonality on microbial diversity patterns could also be linked to seasonal energy production (Manganelli *et al*., 2009; Raes *et al*., 2020). Some taxa, such as *N*. *catalina*, is known to be less tolerant to warm temperatures (Ahlgren *et al*., 2017) and probably thrived better and exhibited higher relative abundance in colder winter waters when the Pacific sector was sampled.

The significant contribution of oxygen in the DistLM analysis (Table S5) indicated that the varying oxygen levels between Pacific and Indian SAMW/SASW could explain the sector specificity of taxa such as SAR324 and Marinimicrobia. A phylogenetic tree of the SAR324 sequences to those from other published studies found that the Pacific SAMW/SASW SAR324 were closely related to Clade II SAR324 (Fig. S9). Clade II SAR324 is known to be restricted to low‐latitude, deep waters with lower oxygen availability, congruent with the lower oxygen levels in Pacific SAMW/SASW (Figs. S1, S2; Table S1) that are linked to the oceanic circulation and sub‐Antarctic regions of the Pacific being a water mass formation region (Sloyan and Rintoul, 2001; Talley *et al*., 2016).

### Sectorial variation of the deep‐water community

While PERMANOVA indicated that sector structured a smaller proportion of the prokaryote community variation below the MLD (Table S3B), several dominant bacterial and archaeal taxa were observed to exhibit sectorial variability. Sectorial variability was distinct at family level for Bacteroidetes, where there were higher relative abundances of Bacteroidetes within Indian AABW (Fig. S7A; up to 41% of all bacteria in some samples) with a substantially larger proportion of *Polaribacter* at higher latitudes (>60°S; up to 32% of all Bacteroidetes) compared to Pacific AABW which had a lower proportion (up to 9.6% of all bacteria) and varying composition of Bacteroidetes (Figs. 3A and 4C, Fig. S8). For the archaea, sectorial variation was also observed in the AABW where there were substantially higher proportions of *Nitrosopumilus* in the Indian sector (up to 81% of all archaea) compared to the Pacific (up to 19% of all archaea) where *Nitrosopelagicus* was more dominant (up to 76% of all archaea) (Figs. 3 and 4D, Fig. S7B; see also pair‐wise SIMPER, Table S4). While PCA indicated a strong sectorial variability of the environmental parameters (Fig. S10C) that initially suggested they could explain the observed sectorial community variation, DistLM showed that environmental and geographical predictors combined only explained 30% and 40% of the bacterial and archaeal community variation respectively (Table S5). Spatial distance alone was also not a key factor driving community variation. This was illustrated by distance–decay plots (Fig. S11C‐D) showing that both AABW bacteria and archaea had weak to no correlation with geographical distance compared to a relatively strong negative correlation observed between surface prokaryotes with geographical distance (Fig. S11A‐B; bacteria: *ρ* = −0.69, *p* < 0.001; archaea: *ρ* = −0.39, *p* < 0.001).

Historical contingencies (Fukami, 2015) could also contribute to the sectorial variability of the deep‐water prokaryotes. For instance, the sector‐specific higher abundance of *Polaribacter* (Fig. 4C, Fig. S7A), a dominant surface‐Antarctic coastal Bacteroidetes that is commonly associated with the degradation and remineralization of phytoplankton blooms (Abell and Bowman, 2005a; Straza *et al*., 2010; Landa *et al*., 2018; Kim *et al*., 2019) in AABW is very likely to be linked to/transmitted from the flourishing summer surface community (that is entrained to the abyss during AABW formation). It should be noted that varying proximity between Indian and Pacific AABW sampling sites from their bottom water formation regions may also influence the communities that are entrained from surface waters during the bottom water formation process.

### Deep‐water prokaryote communities display high beta‐diversity within the same sector

Prokaryote communities from deep water masses within the same sector also displayed high heterogeneity, particularly for the bacterial community as clearly indicated by greater dispersion in the nMDS plot (Fig. 2A). The high dispersion amongst the deep water mass samples was corroborated by average Bray–Curtis similarity values of bacterial community in the respective deep water masses (AABW, LCDW, UCDW; with each sector considered separately) which ranged between 21% and 34% compared to those of samples within epipelagic water masses (SAMW, SASW, CSW, SICW, AAIW) ranging between 33% and 72% (Table S6). Archaeal beta‐diversity was lower compared to the bacterial community between deep water mass samples (Fig. 2B); average similarity of archaeal communities from deep water masses was 39%–52%, while epipelagic archaeal communities had similarity values ranging between 35% and 67% (Table S6).

The community heterogeneity was especially apparent in the AABW bacterial community from the Indian sector, with only 21% community similarity between sampling sites (Table S6) and showed a spatially inconsistent (patchy) distribution (Fig. 2A) that was not distance related (Fig. S11C). This high and inconsistent heterogeneity could be attributed to several mechanisms. Large proportions of the microbial community in the deep ocean are known to be sustained by sinking, microbially colonized organic C particles generated in the epipelagic (Herndl and Reinthaler, 2013), and as a result, the deep ocean microbial community structure can be influenced by variations in the surface microbial assemblage (Baltar and Arístegui, 2017), especially the particle‐attached surface community (Mestre *et al*., 2018). However, varying particle sinking speeds (Ploug *et al*., 2008) and the influence of trophic and functional structure of the surface ocean on the particles delivered results in intermittent and heterogeneously distributed particles colonizing the deep ocean community (Buesseler *et al*., 2009; Smith Jr. *et al*., 2018; Acinas *et al*., 2021), which may explain the patchy, high beta‐diversity observed (Hewson *et al*., 2006). Additionally, while advection has also been shown to influence beta‐diversity (Wilkins *et al*., 2013a) by increasing dispersal, deep water currents are generally slow (~43 m per day) (Haine *et al*., 1998) and, alone, will probably not effectively support long‐distance homogenizing dispersal of deepwater microbial communities, leading to heterogeneity observed between deep water sites.

### Archaeal community composition reveals surprising depth distribution patterns

Several noteworthy depth distribution patterns were observed within the archaeal community. First was the abundant presence of the Crenarchaeota phylum (formerly Thaumarchaeota) in water masses across depths and sectors (15%–100%; Fig. 3B, excluding values in SICW), supporting a growing number of studies that Crenarchaeota do not only exhibit increasing abundances with depth but are also abundant in the euphotic zone, especially in polar oceans (Shiozaki *et al*., 2016; Sintes *et al*., 2016). Additionally, Crenarchaeota, which was previously found to be seasonally abundant only in Antarctic winter surface waters (Murray *et al*., 1998; Church *et al*., 2003; Kalanetra *et al*., 2009; Grzymski *et al*., 2012) were found to be abundant (mainly genus *Nitrosopumilus*) in both summer (Indian sector) and winter (Pacific sector) AASW in this study (Fig. 3B). Second was the high relative abundance (up to 76%) and persistence of several *Nitrosopelagicus*‐classified zOTUs across the deep (UCDW, LCDW and AABW) water masses (except AABW of the Indian sector), in contrast to the low relative abundance of this clade within epipelagic water masses (Fig. 3B). Current literature suggests *Nitrosopelagicus* is primarily found in shallow waters, and apart from another *Nitrosopelagicus*‐related Crenarchaeota sequence detected at 800 m of the South Atlantic Subtropical Gyre (Rinke *et al*., 2013), no other *Nitrosopelagicus* or affiliated sequences have been detected or linked to known deep‐water Crenarchaeota clades (Sintes *et al*., 2013; Santoro *et al*., 2017). Interestingly, each of the lower mesopelagic and bathy‐abyssopelagic water masses had their own specific taxonomic composition of *Nitrosopumilus*, which was consistently observed across both sectors (Fig. 4D). This composition was distinct from those in the epipelagic water masses that were dominantly inhabited by *Nitrosopumilus* sp. 2730325 (Fig. 4D).

Similar to the distribution patterns of the Crenarchaeota, we also found the phylum Thermoplasmatota (formerly Marine Group II, referred to as MGII in this article) to be abundant across depths and sectors (9%–84%) (Fig. 3B). Exceptions were in the Indian CSW where Thaumarchaeota were dominant, and Indian AASW where there was a mixed dominance of Thaumarchaeota in some sites and MGII in others (Fig. 3B). This was in contrast to the common perceptions of MGII as a surface ocean dweller (Zhang *et al*., 2015). Mixed patterns have been observed in previous work within the WAP – Church *et al*. (2003) found low numbers of MGII throughout the water column in both summer and winter samplings, while Signori *et al*. (2014) found MGII to be enriched in deeper summer waters. MGII abundances have also been reported to be invariable by depth within the Atlantic region (Teira *et al*., 2004; Herndl *et al*., 2005; Santoro *et al*., 2019). While MGII relative abundance was relatively invariable throughout the water column, the taxonomic composition of MGII was distinct in the surface and deep water masses, with MGIIb‐O2, MGIIbO3 and UBA59 prevalent in surface/above MLD water masses, while MGIIb‐O1 and Thalassarchaeum were the more common MGII found in deep/below MLD water masses (Fig. 3B, Fig. S7B). The dominance of different Crenarchaeota and Thermoplasmatota groups in the epipelagic versus those below the epipelagic water masses, especially at finer (genus to zOTU) taxonomic levels suggests niche specialization, metabolic or functional diversification of closely related groups, in‐line with the findings of some earlier studies, e.g. Francis *et al*. (2005), Hallam *et al*. (2006), Luo *et al*. (2014) and Rinke *et al*. (2019).

### Concluding remarks

In summary, this article presented a comprehensive dataset that allowed us to explore in‐depth the prokaryote community diversity simultaneously across a large latitudinal, longitudinal and depth scale. The large spatial and depth coverage of this dataset allowed us to show that oceanographically similar water masses, whether in the epi‐, meso‐ or bathypelagic can exhibit variations in community composition of key prokaryote taxa across different sectors of the SO. The high prokaryote diversity and lesser expected sectorial variability in the meso‐ and bathypelagic water masses is likely linked to vertical connectivity with microbial and ecosystem activity occurring in the different epipelagic water masses (and its varying environmental conditions) it is linked to. Future studies can explore how this high diversity and heterogeneous distribution is linked to vertical connectivity and varying composition of organic matter due to differing activities in the surface.

## Experimental procedures

### Study region and sampling description

Seawater samples were collected during three voyages in 2016. The first two voyages were on the R/V Investigator: the Heard Earth‐Ocean‐Biosphere Interactions (HEOBI) voyage (8th January–27th February, 2016; austral summer) and Leg 1 of the GO‐SHIP P15S repeat hydrographic transect (http://www.go-ship.org; 26th April to 26th May, 2016; austral winter). The third voyage was on the Aurora Australis icebreaker: the Kerguelen Axis (K‐Axis) voyage (11th January–24th February 2016; austral summer; Fig. 1A). As our sampling was opportunistic, we could not control for a common sample collection season between sectors. Details on sample collection devices and sensors are in supplementary methods.

In all voyages, samples were collected at three to eight different depths from surface to bottom waters from 66°S to 39.5°S at longitude ranges of 71–99°E and 170–174°W (Fig. 1B and C). The depths were selected to target samples from the surface (SFC), chlorophyll maximum depth (CMX), MLD, below MLD, 500, 1000, 2000 m, and the bottom depth. For CMX and MLD, sampling depths were selected based on temperature, salinity, DO and fluorescence signal profiles. In total, 223 samples were considered within this study; 64 samples were from GO‐SHIP P15S (Pacific sector), 78 samples were from HEOBI (Indian sector) and 81 samples were from K‐Axis (Indian sector) (Table S1).

The water mass where samples were collected were identified following temperature and salinity criteria ranges in Emery (2003) and with reference to Pardo *et al*. (2012). The nine water masses identified are listed in Fig. 1, with detailed definitions listed in supplementary methods. The number of samples collected from each water mass is detailed in Table S2.

### Hydrological variable measurements and nutrient analyses

Samples for the dissolved inorganic nutrients (silicate [Si], phosphate [PO_4_^3−^], nitrate [NO_3_^−^] and nitrite [NO_2_^−^]) analyses were assayed by the CSIRO Hydrochemistry team on a Bran + Luebbe AA3 HR segmented flow analyzer following standard spectrophotometric methods (Hansen and Koroleff, 2009). Detailed methodology, detection limits and links to access the physical, biogeochemical, nutrient and metadata discussed here (temperature, salinity, DO, nitrate + nitrite [NOx], NO_2_^−^, PO_4_^3−^ and Si) are available in supplementary methods.

### DNA isolation, amplification, sequencing and sequence data analyses

Two litres of seawater was filtered through 0.22 μm pore size polyethersulfone Sterivex™ filter cartridges (Millipore, Darmstadt, Germany) for each sample and immediately stored at −80°C until DNA extraction. Each microbial sample was issued a unique ID (BPA ID) from the Australian National Data Service (Table S1). DNA was isolated using a modified phenol:chloroform:isoamyl based extraction protocol of the DNeasy PowerWater Sterivex Kit (MoBio‐Qiagen, Hilden, Germany) (Appleyard *et al*., 2013) and stored at −20°C.

DNA amplification and sequencing were performed at the Ramaciotti Centre for Genomics (Sydney, Australia). Prokaryote assemblages were surveyed using V1–V3 hypervariable regions of the 16S rRNA gene amplified using primers 27F–519R (bacteria) (Lane *et al*., 1985; Lane, 1991) and A2F–519R (Lane *et al*., 1985; DeLong, 1992). Amplicons were sequenced using Illumina's MiSeq dual index 300 bp paired‐end approach (Illumina, San Diego, CA, USA). All data including paired‐end reads (R1, R2), indexed reads (I1, I2) data in .fastq format, and latest sequence read abundance tables are available from the Australian Microbiome Initiative data portal (https://data.bioplatforms.com/organization/about/australian-microbiome).

### Sequence data analysis and accession numbers

All sequences were analyzed as part of the Australian Microbiome Initiative as previously described by Brown *et al*. (2018). Detailed methods used for sequence data analysis and taxonomic classification are in supplementary methods. zOTU data with single nucleotide variation between zOTUs were used to enable data analysis at the highest possible phylogenetic variation. zOTU tables were rarified to 10 000 reads per sample for subsequent statistical analysis using the ‘sub.sample’ command in MOTHUR. Genomic datasets associated with this study are accessible from NCBI Bioprojects numbers IDs PRJNA385736 and PRJNA509756. Sequence accession numbers are listed in Table S1.

### Statistical analyses, diversity indices and correlation with environmental parameters

Alpha diversity matrices were calculated using the ‘scikit‐bio’ and *‘*ecopy’ (Lemoine, 2015) packages on Python 3.6.1. Rarefaction curves were generated using the ‘rarecurve’ function in the R package ‘vegan’ (Oksanen *et al*., 2018). Good's coverage was calculated using ‘scikit‐bio’ based on the formula 1‐(*F*_1_/*N*), where *F*_1_ is the number of singletons and *N* is the sum of zOTU abundances. Beta‐diversity measures were used to assess spatial turnover patterns of prokaryote communities between water masses. We used PRIMER [v7.0.18; Clarke and Gorley, 2015] to generate Bray–Curtis taxonomic similarity matrices following fourth‐root transformation of zOTU abundances and visualized community differences with nMDS plots. Community variations were assessed separately for bacterial and archaeal communities below and above the mixed layer, based on either water mass or oceanographic sector (i.e. Indian or Pacific) with permutation multivariate analysis of variance (PERMANOVA) in PRIMER (Anderson *et al*., 2008). Two‐way crossed PERMANOVA tests were used to assess effects of factor interaction (water mass × sector) on community variation with marginal test of terms and 9999 permutations of pseudo‐F ratios. Average contribution of individual zOTUs (then grouped by phyla, family or genera) to Bray–Curtis dissimilarity between pairs of water mass × sector was calculated using the SIMPER routine with an 80% cumulative contribution cut‐off value (PRIMER). Average Bray–Curtis similarity percentages of the prokaryote community within each water mass were identified using the ANOSIM routine (PRIMER).

DistLM was used to assess the relationship between environmental [depth (pressure), oxygen, temperature, salinity, silicate, nitrite, NOx, phosphate, day‐length] and geographical (latitude and longitude) predictors that best explained the observed prokaryote beta‐diversity. DistLM models were computed using Bray–Curtis similarity matrices based on fourth root transformed bacterial and archaeal relative abundances with transformed and normalized environmental and geographical variables (see above) in PRIMER (Clarke and Gorley, 2015). Model selection procedures and criteria are detailed in supplementary methods. Models were created for all samples and subsets of surface and AABW samples. Surface samples analyzed include all samples taken from depths <20 m from all stations.

Effects of geographical distance on community variation were assessed with distance–decay plots. Methods to generate geographical distance in the distance–decay correlation plots (Fig. S11) are explained in supplementary methods.

## Author Contributions

S.L.S.S., E.J.R. collected GO‐SHIP samples and executed experiments; L.J.C. collected K‐Axis samples; T.W.T. collected HEOBI samples. E.J.R., L.B. and J.v.d.K came up with a preliminary experimental design; A.B., J.v.d.K, S.S.L.S. analyzed the bioinformatics; S.S.L.S. executed statistical analysis and DistLM models, P.C.P. and B.M.S. provided physical oceanography advice. S.L.S.S. led the write‐up; all authors contributed to interpreting data, reviewing and refining the article.

## Supporting information


**Appendix** **S1**: Supplementary methods
**Fig. S1**. A. Temperature salinity curves along the P15S transect (Pacific sector) overlayed with corresponding dissolved oxygen values, illustrating the main water masses (labelled with its corresponding acronym). Contour lines indicate potential density anomaly values. Vertical distributions of B. oxygen, C. salinity, D. nitrate + nitrite (NO_x_), E. nitrite, F. phosphate and G. silicate along leg 1 of the P15S transect.
**Fig. S2.** A. Temperature salinity curves along the HEOBI and K‐Axis voyages (Indian sector) overlayed with corresponding dissolved oxygen values, illustrating the main water masses (labelled with its corresponding acronym). Contours lines indicate potential density anomaly values. Vertical distributions of B. oxygen, C. salinity, D. nitrate + nitrite (NO_x_), E. nitrite, F. phosphate and G. silicate along the HEOBI and K‐Axis voyages.
**Fig. S3**. Rarefaction curves of observed A. bacteria and B. archaea species. Samples were subsampled to a depth of 10,000 sequences as indicated by grey dotted lines prior to subsequent analyses.
**Fig. S4.** Rarified mean alpha‐diversity indices for prokaryotes within different SO water masses. A. Richness and Chao1 index; B. Pielou's evenness and Shannon‐Weaver index. Water masses acronyms are as shown in Fig. 1.
**Fig. S5**. Plot of bacterial and archaeal community A. Richness against dissolved oxygen and B. Pielou's evenness against dissolved oxygen.
**Fig. S7.** Shade plots indicating overall relative abundance (at phylum/class level) of A. bacteria and B. archaea detected, within each water mass and sector they were sampled from. Detailed charts of the main families of C. Gammaproteobacteria, D. Alphaproteobacteria, E. Bacteroidetes and F. main species/zOTUs of the archaeal *Nitrosopumilus* genus within each water mass‐sector are also shown. ‘Other phyla’ or ‘Other genera’ are bacterial phylum or archaeal genera with overall mean relative abundances of <0.5% across all water masses. ‘Unclassified archaea’ include archaeal zOTUs that were classified with bootstrap confidence values <50%. Water mass name acronyms and water mass‐sector symbols are as indicated in Fig. 1 and Fig. 2 legends.
**Fig. S8**. Relative abundances of Bacteroidetes within the AABW samples. Samples are sorted by sector and latitude. Sample ID – Sector; Water Mass; Voyage.Latitude.Depth (m). P – Pacific sector; I – Indian sector; G – GO‐SHIP; KX – K‐Axis; H – HEOBI
**Fig. S9**. Phylogenetic tree of abundant SAR324 zOTU sequences from Pacific SAMW and SASW samples clustered with other SAR324 sequences with identified clades. Sequences with the prefix ‘AMD’ in blue are sequences from this study and are not specific to either SAMW or SASW water masses only.
**Fig. S10**. PCA ordination of the environmental and geographical properties of (A) all samples (B) epi‐ and mesopelagic samples and (C) bathy‐ and abyssopelagic samples considered within this study, grouped according to the water masses they were assigned to. Water mass name acronyms are as indicated in Fig. 1.
**Fig. S11.** Distance‐decay plots of Bray–Curtis similarity values between (A‐B) surface water and (C‐D) AABW prokaryote samples versus geographical distance between the samples.
**Table S2**. Alpha diversity matrices for bacterial and archaeal 16S rRNA gene sequences within the different Southern Ocean water masses.
**Table S6.** Average Bray–Curtis similarity percentages of the prokaryote community within the various water masses.
**Table S7**. Pearson correlation of environmental variables considered within this study. ^+^Surface samples analysed include all samples taken from depths <20 m from all stations.

**Fig. S6.** **(a)** Full charts for relative abundance of major bacteria detected from the different water masses at phylum/class level, showing each of the 223 samples on the x‐axis. Samples are arranged from high to low latitude in each water mass from the Pacific (**P**) followed by Indian (**I**) sector. Detailed charts of the main families within **(b)** Gammaproteobacteria, **(c)** Alphaproteobacteria and **(d)** Bacteroidetes is also shown. The full chart for archaeal relative abundance is shown in **(e)**, and a detailed chart of the main *Nitrosopumilus* species/zOTUs is shown in **(f)**.

**Table S1.** List of samples, sampling information and contextual data for all samples collected within this study.

**Table S3.** **A**: Permutational multivariate analysis of variance using distance matrices (PERMANOVA) on Bray‐Curtis resemblance matrices of fourth root transformed bacterial and archaeal zOTU abundance data (number of permutations = 9999). *p ≤ 0.0001, **p ≤ 0.01
**Table** **S3** **B**: Crossed two‐factor permutational multivariate analysis of variance using distance matrices (PERMANOVA) on Bray‐Curtis resemblance matrices of fourth root transformed bacterial and archaeal zOTU abundance data (number of permutations = 9999). *p ≤ 0.0001, **p ≤ 0.01
**Table** **S3** **C**: PERMDISP on Bray‐Curtis resemblance matrices of fourth root transformed bacterial and archaeal zOTU abundance data (number of permutations = 9999). *p ≤ 0.0001, **p ≤ 0.01
**Table** **S3** **D**: Results from PERMANOVA pairwise tests for the interaction term of Sector x Water Mass in bacterial and archaeal communities. Tests were based on Bray‐Curtis resemblance matrices of fourth root transformed bacterial and archaeal zOTU abundance data (number of permutations = 9999). *p ≤ 0.0001, **p ≤ 0.01

**Table S4.** SIMPER analysis of the A. bacterial and B. archaeal community, grouped by phylum/class, family and genus, indicating the contribution of each phylum/class/family/genus to community dissimilarity between the different water masses in the Pacific sector and the same corresponding water mass in the Indian sector. The analysis included higher contributing zOTUs up to a cumulative cut‐off percentage of 80%.

**Table S5.** Proportion of variation explained by individual predictor variations within Distance‐based linear modelling (DistLM) marginal tests. *Surface samples analysed include all samples taken from depths <20 m from all stations. ‘‐’ denotes that variable was not included in model due to high co‐linearity (Pearson's r > |0.85|) with another variable that contributed to explaining more significant proportions of community variation. ** indicate non‐significant *p*‐values (*p* > 0.05); nPerm = 9999
