# Supplementary material for: Biogeography of Southern Ocean prokaryotes: a comparison of the Indian and Pacific sectors
Source: Environ Microbiol. 2022 Feb 16;24(5):2449–66. doi: 10.1111/1462-2920.15906 (PMC9303206; doi:10.1111/1462-2920.15906)
Supplement: Supplementary file 1 — Appendix S1: Supplementary methods Fig. S1. A. Temperature salinity curves along the P15S transect (Pacific sector) overlayed with corresponding dissolved oxygen values, illustrating the main water masses (labelled with its corresponding acronym). Contour lines indicate potential density anomaly values. Vertical distributions of B. oxygen, C. salinity, D. nitrate + nitrite (NOx), E. nitrite, F. phosphate and G. silicate along leg 1 of the P15S transect. Fig. S2. A. Temperature salinity curves along the HEOBI and K‐Axis voyages (Indian sector) overlayed with corresponding dissolved oxygen values, illustrating the main water masses (labelled with its corresponding acronym). Contours lines indicate potential density anomaly values. Vertical distributions of B. oxygen, C. salinity, D. nitrate + nitrite (NOx), E. nitrite, F. phosphate and G. silicate along the HEOBI and K‐Axis voyages. Fig. S3. Rarefaction curves of observed A. bacteria and B. archaea species. Samples were subsampled to a depth of 10,000 sequences as indicated by grey dotted lines prior to subsequent analyses. Fig. S4. Rarified mean alpha‐diversity indices for prokaryotes within different SO water masses. A. Richness and Chao1 index; B. Pielou's evenness and Shannon‐Weaver index. Water masses acronyms are as shown in Fig. 1. Fig. S5. Plot of bacterial and archaeal community A. Richness against dissolved oxygen and B. Pielou's evenness against dissolved oxygen. Fig. S7. Shade plots indicating overall relative abundance (at phylum/class level) of A. bacteria and B. archaea detected, within each water mass and sector they were sampled from. Detailed charts of the main families of C. Gammaproteobacteria, D. Alphaproteobacteria, E. Bacteroidetes and F. main species/zOTUs of the archaeal Nitrosopumilus genus within each water mass‐sector are also shown. ‘Other phyla’ or ‘Other genera’ are bacterial phylum or archaeal genera with overall mean relative abundances of <0.5% across all water masses. ‘Unclassified a [file EMI-24-2449-s006.docx]

# Supporting Information

Biogeography of Southern Ocean prokaryotes: a comparison of the Indian and Pacific sectors

Swan L.S. Sow^1,2,†,*^, Mark V. Brown^3^, Laurence J. Clarke^1,5^, Andrew Bissett^2^, Jodie van de Kamp^2^, Thomas W. Trull^2^, Eric. J. Raes^2^, Justin R. Seymour^4^, Anna R. Bramucci^4^, Martin Ostrowski^4^, Philip W. Boyd^1^, Bruce E. Deagle^5,6^, Paula C. Pardo^2^, Bernadette M. Sloyan^2^ and Levente Bodrossy^2^

1. Institute for Marine and Antarctic Studies, University of Tasmania, Hobart, Tasmania, Australia

2. Oceans and Atmosphere, Commonwealth Scientific and Industrial Research Organisation, Hobart, Tasmania, Australia

3. School of Environmental and Life Sciences, University of Newcastle, New South Wales 2308, Australia

4. Climate Change Cluster, University of Technology Sydney, 2007, New South Wales, Australia.

5. Australian Antarctic Division, Channel Highway, Kingston, Tasmania 7050, Australia

6. National Collections & Marine Infrastructure, Commonwealth Scientific and Industrial Research Organisation, Hobart, Tasmania, Australia

^†^ Current affiliation: Department of Marine Microbiology and Biogeochemistry, NIOZ Royal Netherlands Institute for Sea Research, Den Burg, The Netherlands

*Corresponding author: [swan.lisan@gmail.com](mailto:swan.lisan@gmail.com) | [swan.sow@nioz.nl](mailto:swan.sow@nioz.nl)

**This file includes:**

- Supplementary figures S1-S5; S7-S11
- Supplementary tables S2; S6-S7
- Supplementary methods

Figure S6 and Tables S1, S3, S4 and S5 are available as separate files.

## Supplementary figures

| **A.**  **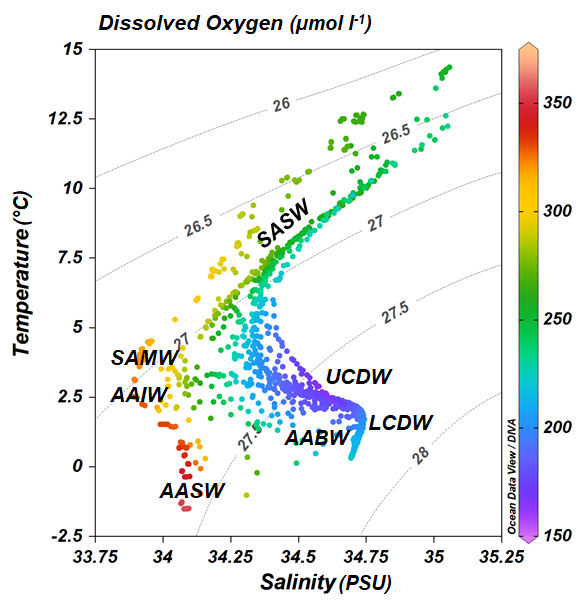** | **B.**  **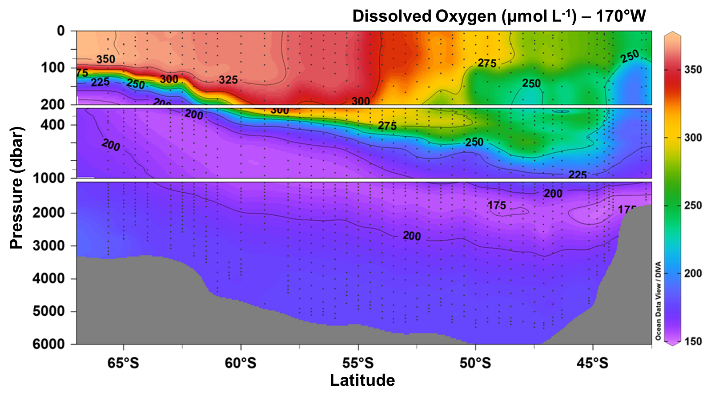** | |
| --- | --- | --- |
|  | **C.**  **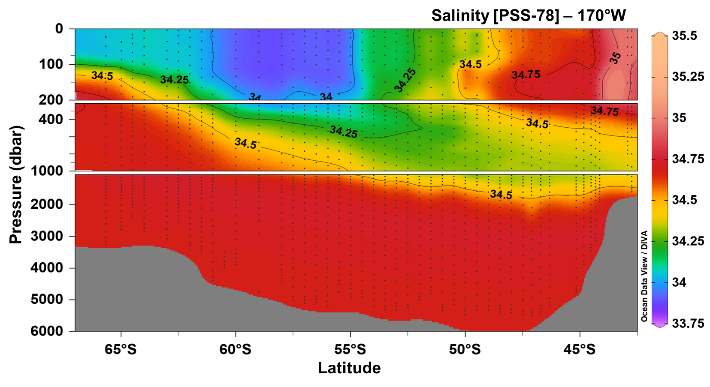** | |
| **D.**  **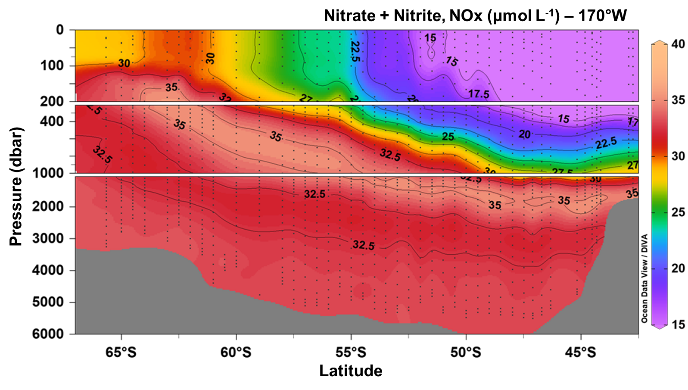** | | **E.**  **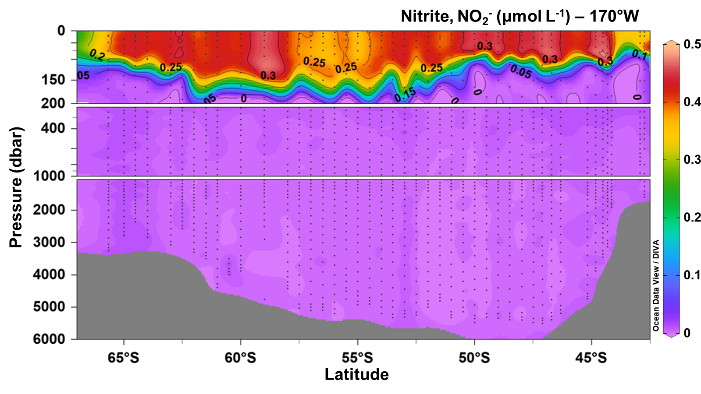** |
| **F.**  **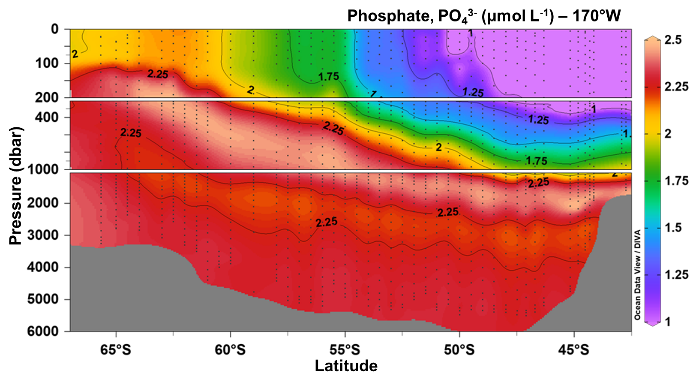** | | **G.**  **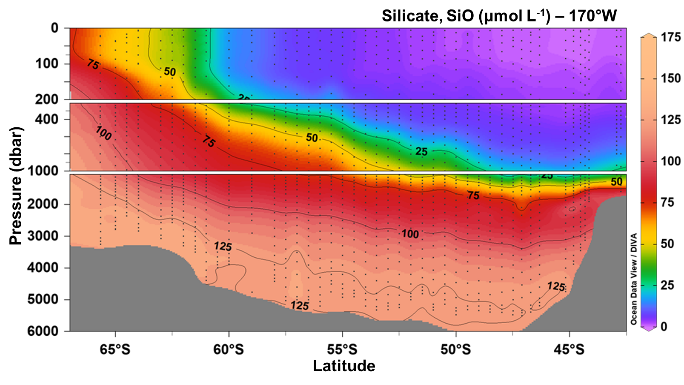** |

**Fig. S1**: A. Temperature salinity curves along the P15S transect (Pacific sector) overlayed with corresponding dissolved oxygen values, illustrating the main water masses (labelled with its corresponding acronym). Contours lines indicate potential density anomaly values. Vertical distributions of B. oxygen, C. salinity, D. nitrate + nitrite (NO_x_), E. nitrite, F. phosphate and G. silicate along leg 1 of the P15S transect.

| **A.**  **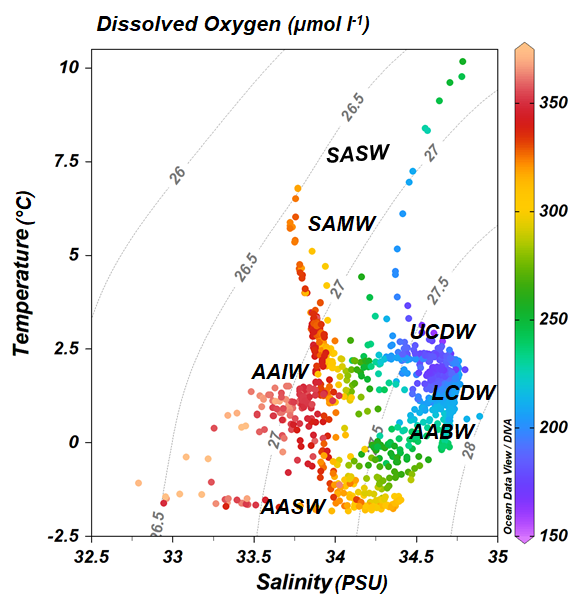** | **B.**  **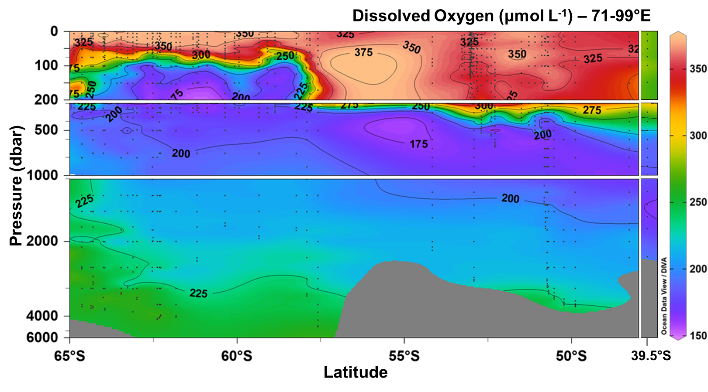** |
| --- | --- |
|  | **C.**  **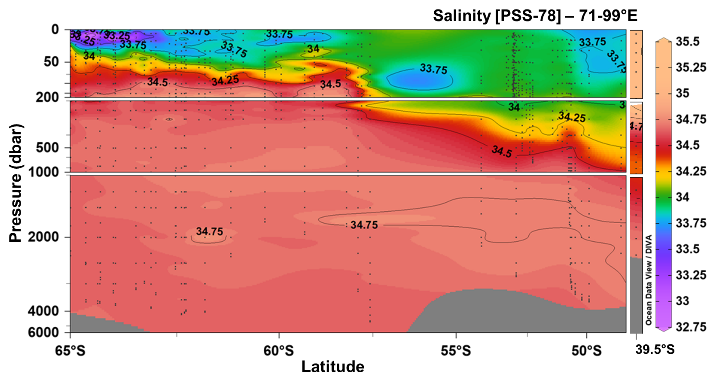** |
| **D.**  **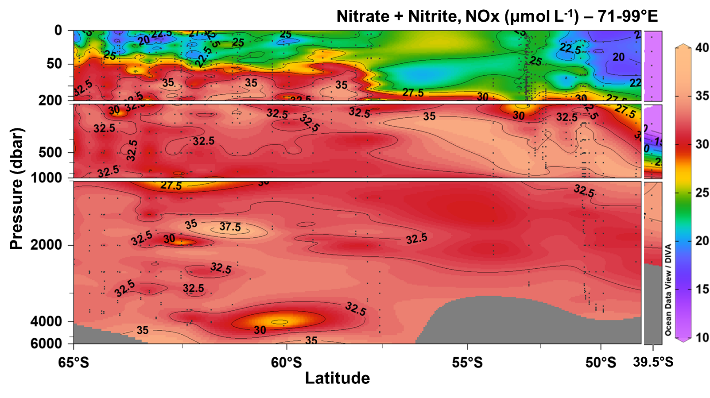** | **E.**  **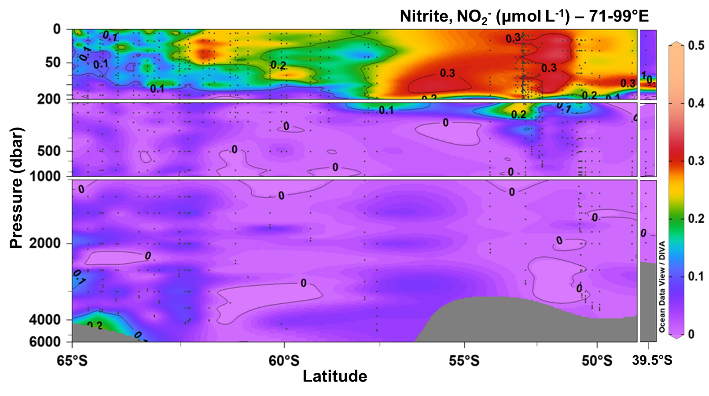** |
| **F.**  **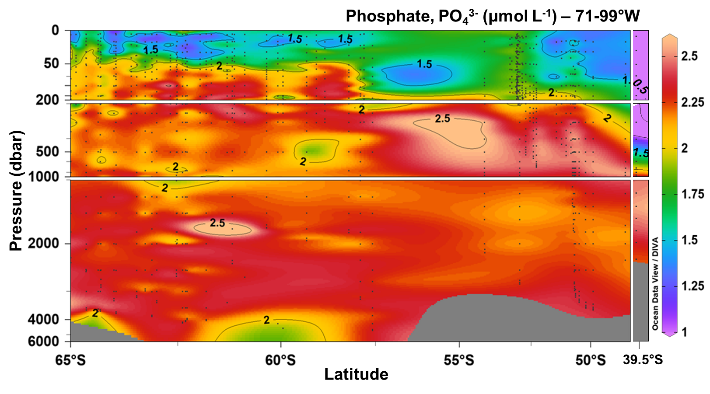** | **G.**  **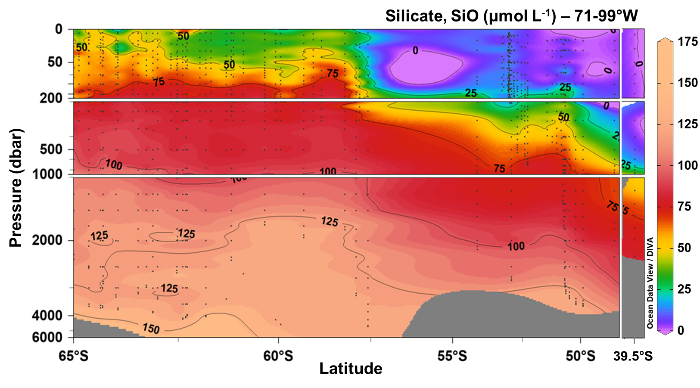** |

**Fig. S2:** A. Temperature salinity curves along the HEOBI and K-Axis voyages (Indian sector) overlayed with corresponding dissolved oxygen values, illustrating the main water masses (labelled with its corresponding acronym). Contours lines indicate potential density anomaly values. Vertical distributions of B. oxygen, C. salinity, D. nitrate + nitrite (NO_x_), E. nitrite, F. phosphate and G. silicate along the HEOBI and K-Axis voyages.

| 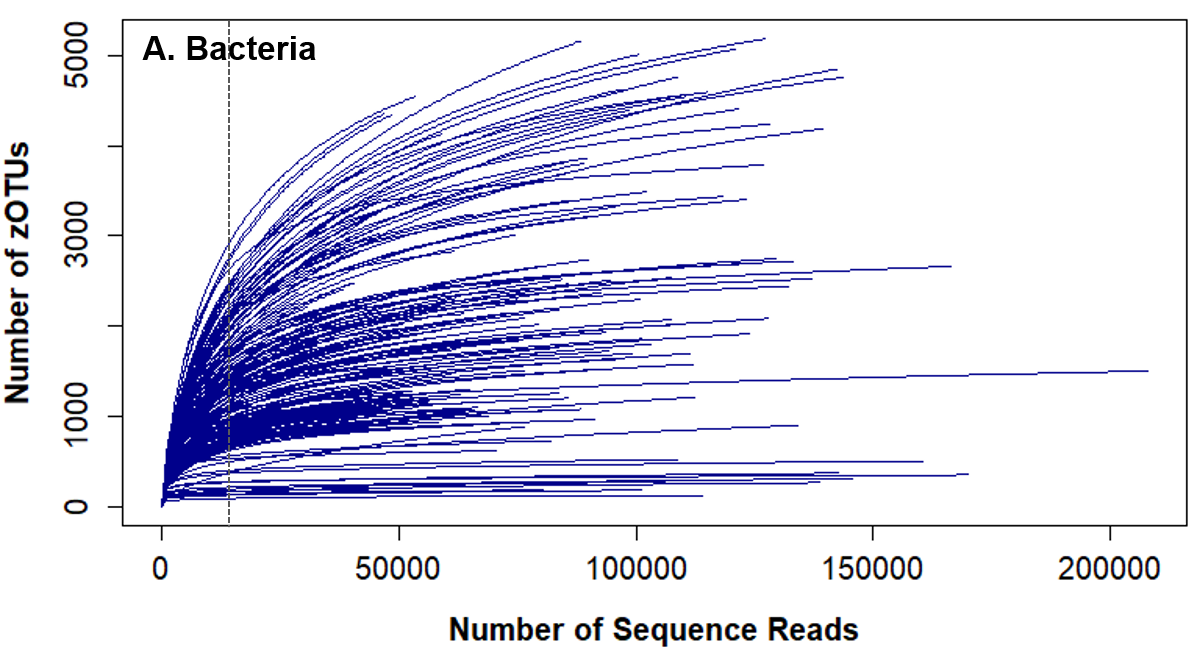 |
| --- |
| 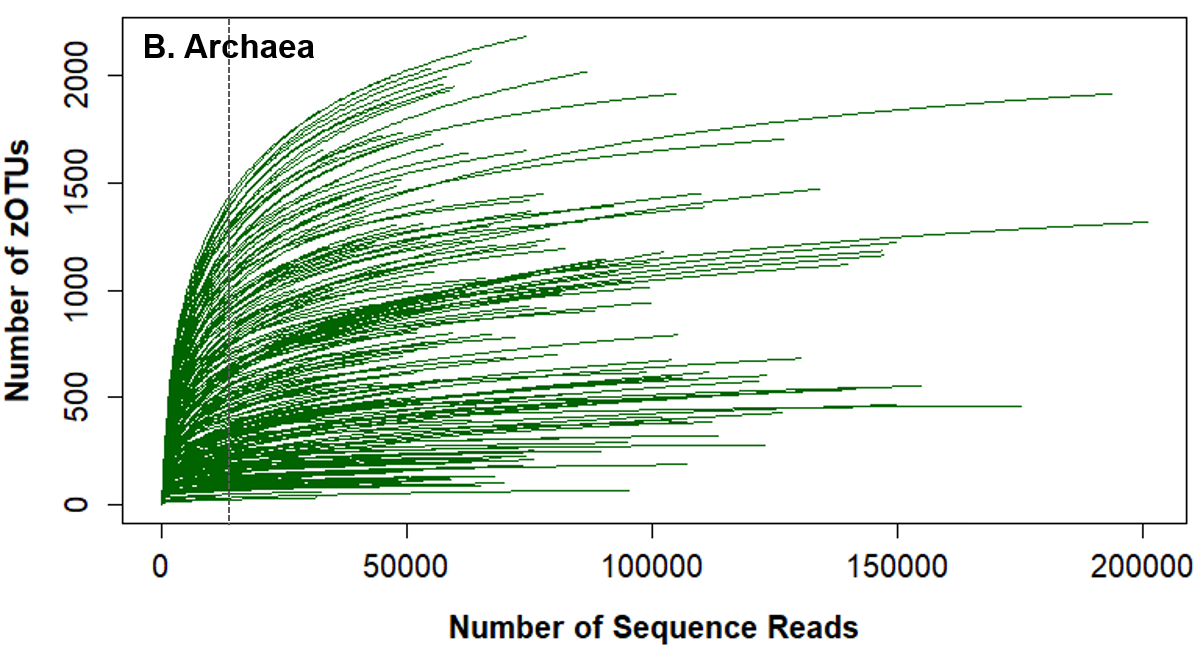 |

**Fig. S3**: Rarefaction curves of observed A. bacteria and B. archaea species. Samples were subsampled to a depth of 10 000 sequences as indicated by grey dotted lines prior to subsequent analyses.

***Oxygen - 71-99°E***

| **A**  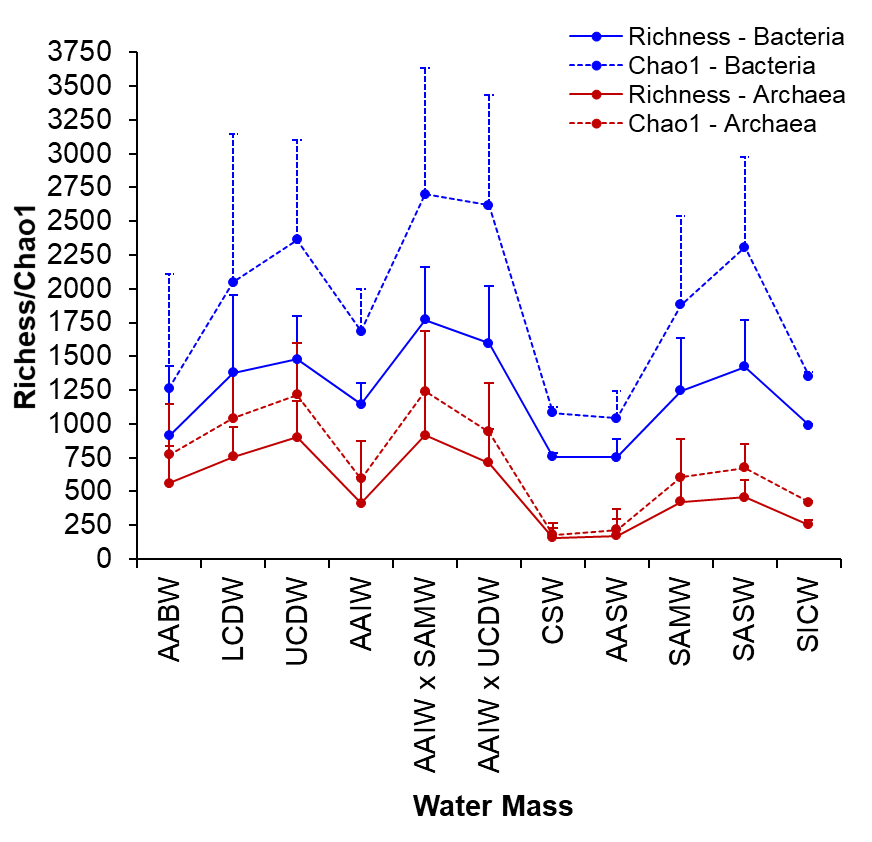 | **B**  **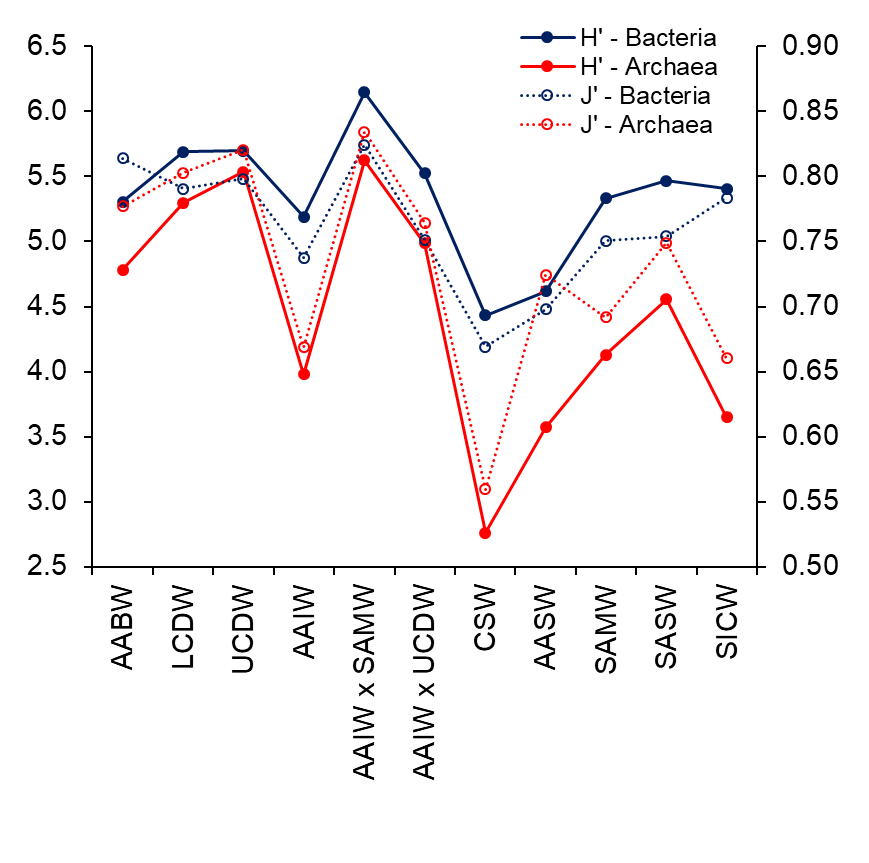** |
| --- | --- |

**Fig. S4**: Rarified mean alpha-diversity indices for prokaryotes within different SO water masses. A. Richness and Chao1 index; B. Pielou’s evenness and Shannon-Weaver index. Water masses acronyms are as shown in Figure 1.

| 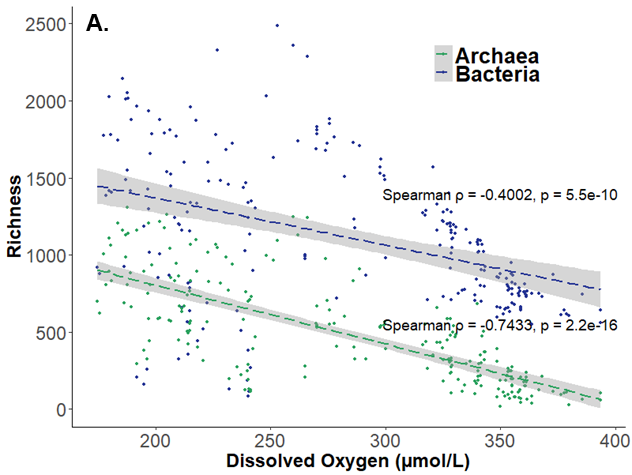 | 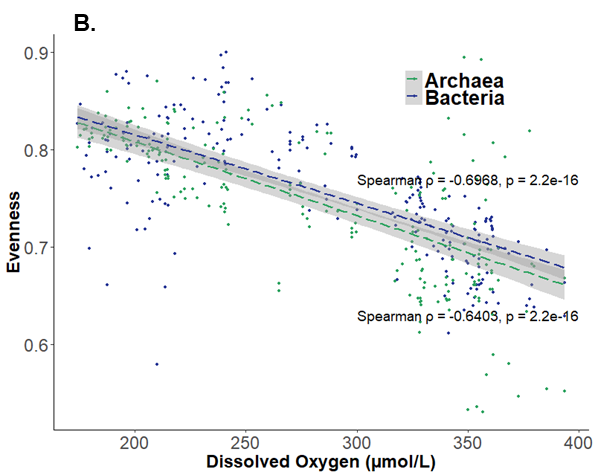 |
| --- | --- |

**Fig. S5:** Plot of bacterial and archaeal community A. Richness against dissolved oxygen and B. Pielou’s evenness against dissolved oxygen.

| **A – All Bacteria**   | **B – All Archaea** **** |
| --- | --- |
| **C – Bacteria: Gammaproteobacteria**   | **D – Bacteria: Alphaproteobacteria**   |

| **E – Bacteria: Bacteroidetes**   | **F – Archaea: *Nitrosopumilus***   |
| --- | --- |

**Fig. S7:** Shade plots indicating overall relative abundance (at phylum/class level) of A. bacteria and B. archaea detected, within each water mass and sector they were sampled from. Detailed charts of the main families of C. Gammaproteobacteria, D. Alphaproteobacteria, E. Bacteroidetes and F. main species/zOTUs of the archaeal *Nitrosopumilus* genus within each water mass_sector are also shown. ‘Other phyla’ or ‘Other genera’ are bacterial phylum or archaeal genera with overall mean relative abundances of <0.5% across all water masses. ‘Unclassified archaea’ include archaeal zOTUs that were classified with bootstrap confidence values < 50%. Water mass name acronyms and water mass-sector symbols are as indicated in Fig.1 and Fig. 2 legends.

**Fig. S8**: Relative abundances of Bacteroidetes within the AABW samples. Samples are sorted by sector and latitude. Sample ID – Voyage.Latitude.Depth(m).CTDId. G – GO-SHIP; KX – K-Axis; H – HEOBI

| 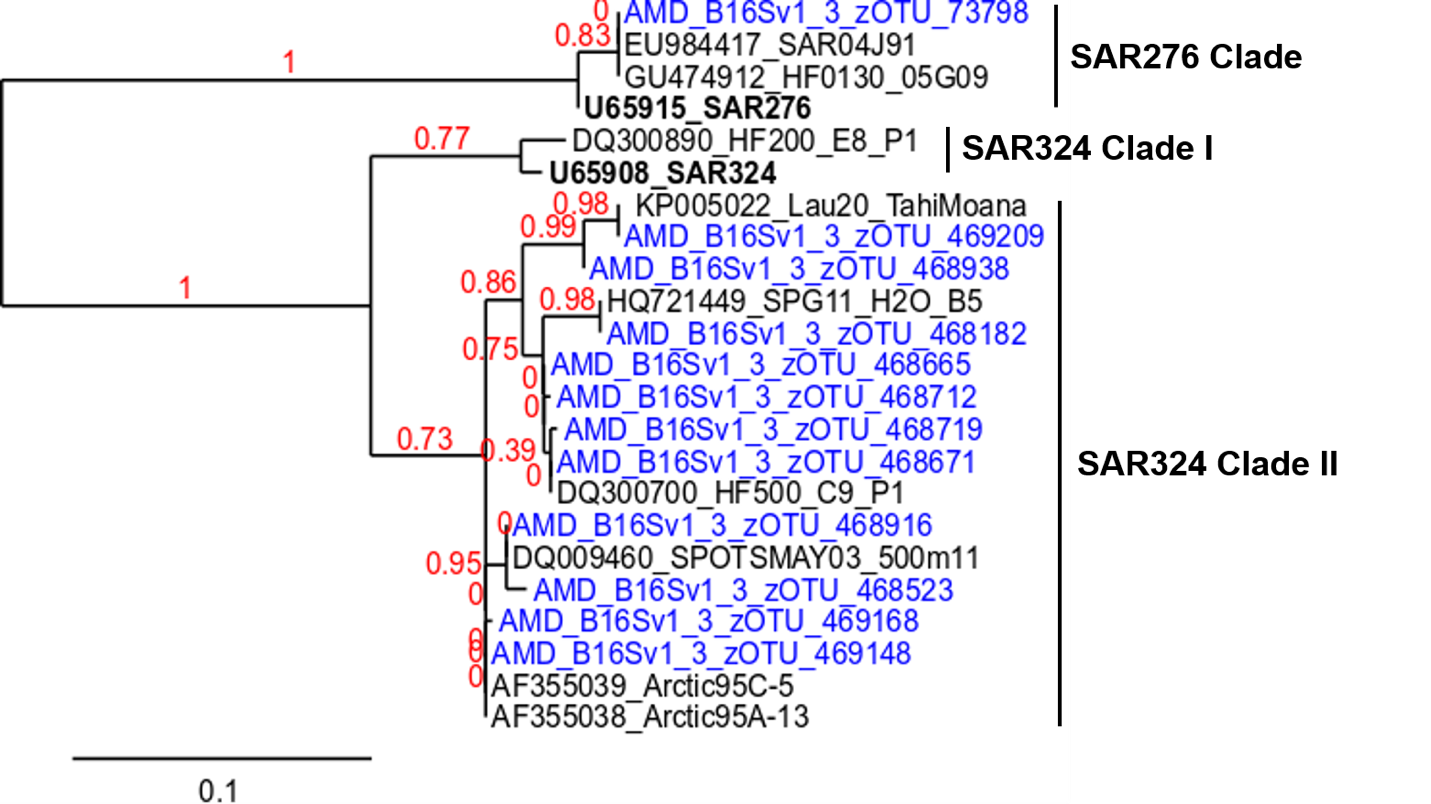 |
| --- |

**Fig. S9**: Phylogenetic tree of abundant SAR324 zOTU sequences from Pacific SAMW and SASW samples clustered with other SAR324 sequences with identified clades. Sequences with the prefix “AMD” in blue are sequences from this study and are not specific to either SAMW or SASW water masses only.

| **A.**  **** | |
| --- | --- |
| **B.**  **** | **C.**  **** |

**Fig. S10:** PCA ordination of the environmental and geographical properties of (A) all samples (B) epi- and mesopelagic samples and (C) bathy- and abyssopelagic samples considered within this study, grouped according to the water masses they were assigned to. Water mass name acronyms are as indicated in Fig. 1

| 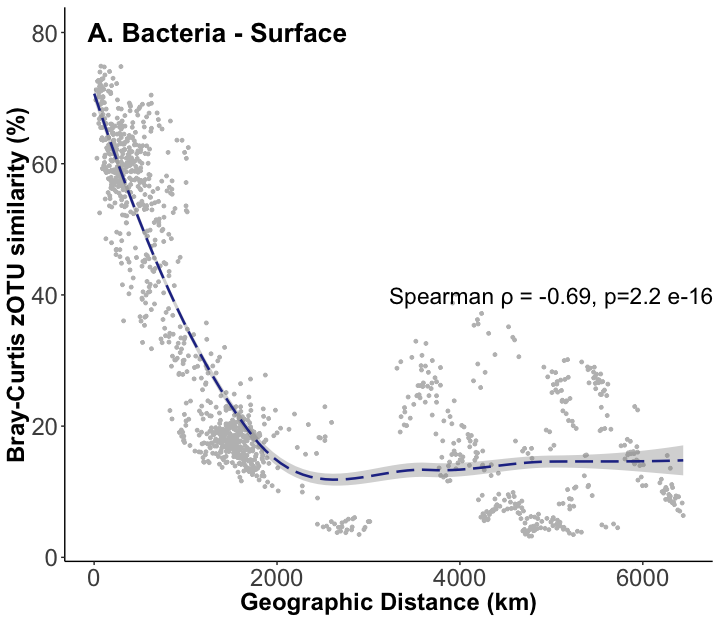 | 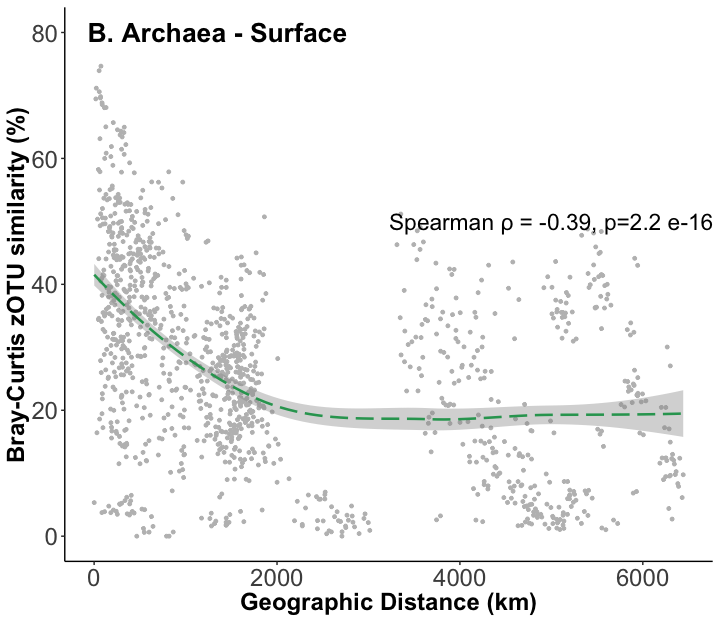 |
| --- | --- |
| 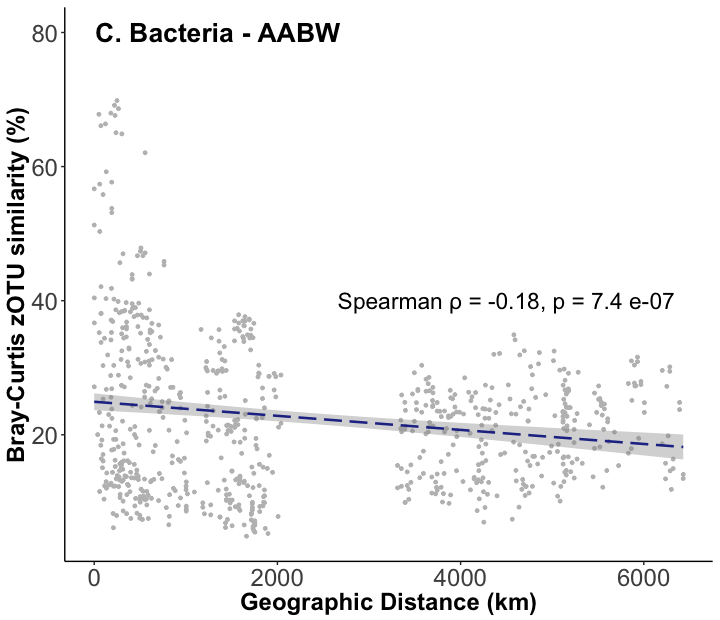 | 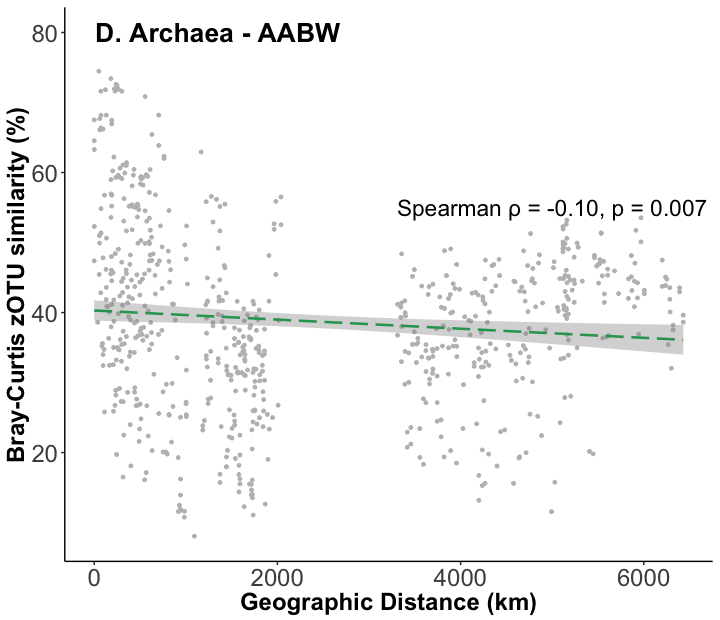 |

**Fig. S11:** Distance-decay plot of Bray-curtis similarity values between (A-B) surface water and (C-D) AABW prokaryote samples versus geographical distance between the sample.

## Supplementary tables

**Table S2**: Alpha diversity matrices for bacterial and archaeal 16S rRNA gene sequences within the different Southern Ocean water masses.

| Water Mass | AABW | LCDW | UCDW | AAIW | AAIW x SAMW | AAIW x UCDW | CSW | AASW | SAMW | SASW | SICW |
| --- | --- | --- | --- | --- | --- | --- | --- | --- | --- | --- | --- |
| Bacteria | | | | | | | | | | | |
| No. of Samples | 39 | 12 | 34 | 21 | 7 | 4 | 6 | 59 | 21 | 17 | 3 |
| Good’s coverage | 0.9683 | 0.9438 | 0.9337 | 0.9534 | 0.9234 | 0.9225 | 0.9703 | 0.9730 | 0.9476 | 0.9348 | 0.9658 |
| Total zOTUs | 10,698 | 6502 | 20,896 | 5303 | 5601 | 3427 | 1515 | 5555 | 7123 | 7020 | 1441 |
| Avg. observed zOTUs/site | 910.5 | 1376.9 | 1480.4 | 1146.2 | 1595.5 | 1772.0 | 757.3 | 751.4 | 1246.6 | 1423.1 | 990.0 |
| Avg. Chao1 | 1264.7 | 2048.4 | 2362.3 | 1686.9 | 2620.1 | 2701.5 | 1082.9 | 1041.0 | 1881.4 | 2309.2 | 1352.2 |
| Avg. Shannon (H’ log_e_) | 5.31 | 5.69 | 5.70 | 5.19 | 5.53 | 6.15 | 4.43 | 4.62 | 5.33 | 5.47 | 5.41 |
| Avg. Simpson (λ’) | 0.018 | 0.015 | 0.017 | 0.038 | 0.035 | 0.009 | 0.045 | 0.041 | 0.028 | 0.032 | 0.016 |
| Avg. gini-Simpson (1-λ’) | 0.982 | 0.985 | 0.983 | 0.962 | 0.965 | 0.991 | 0.955 | 0.959 | 0.972 | 0.968 | 0.984 |
| Avg. Evenness (J’) | 0.81 | 0.79 | 0.80 | 0.74 | 0.75 | 0.82 | 0.67 | 0.70 | 0.75 | 0.75 | 0.78 |
| Avg. Phylogenetic Diversity (φ^+^) | 27.01 | 22.14 | 22.42 | 21.06 | 20.86 | 20.69 | 21.96 | 21.70 | 20.43 | 20.24 | 20.40 |
| Avg. Undersequenced (%) | 28.01 | 32.78 | 37.33 | 32.05 | 34.41 | 39.11 | 30.06 | 27.82 | 33.74 | 38.38 | 26.79 |
| Archaea | | | | | | | | | | | |
| No. of Samples | 38 | 12 | 34 | 21 | 7 | 4 | 6 | 59 | 21 | 17 | 3 |
| Good’s coverage | 0.9817 | 0.9742 | 0.9706 | 0.9848 | 0.9696 | 0.9771 | 0.9971 | 0.9963 | 0.9851 | 0.9838 | 0.9899 |
| Total zOTUs | 4044 | 2734 | 5349 | 2030 | 2556 | 1347 | 440 | 1667 | 2813 | 2437 | 435 |
| Avg. observed zOTUs/site | 561.8 | 758.5 | 900.5 | 412.7 | 916.7 | 712.8 | 157.5 | 173.9 | 424.7 | 458.7 | 254.0 |
| Avg. Chao1 | 770.5 | 1043.1 | 1214.8 | 593.9 | 1240.9 | 941.5 | 178.4 | 217.7 | 606.5 | 673.9 | 421.2 |
| Avg. Shannon (H’ log_e_) | 4.78 | 5.30 | 5.535968 | 3.979172 | 5.62597 | 4.986185 | 2.758836 | 3.571431 | 4.129815 | 4.55572 | 3.648417 |
| Avg. Simpson (λ’) | 0.018 | 0.010 | 0.009 | 0.037 | 0.008 | 0.018 | 0.105 | 0.054 | 0.034 | 0.0192 | 0.043 |
| Avg. gini-Simpson (1-λ’) | 0.981 | 0.989 | 0.991 | 0.963 | 0.992 | 0.982 | 0.895 | 0.945 | 0.966 | 0.981 | 0.957 |
| Avg. Evenness (J’) | 0.78 | 0.80 | 0.82 | 0.67 | 0.83 | 0.76 | 0.56 | 0.72 | 0.69 | 0.75 | 0.66 |
| Avg. Phylogenetic Diversity (φ^+^) | 15.03 | 14.71 | 14.68 | 15.01 | 14.74 | 14.73 | 15.78 | 15.87 | 14.97 | 14.96 | 15.24 |
| Avg. Undersequenced (%) | 27.08 | 27.29 | 25.88 | 30.50 | 26.13 | 24.30 | 11.72 | 20.12 | 29.98 | 31.94 | 39.70 |

**Table S6:** Average Bray-Curtis similarity percentages of the prokaryote community within the various water masses

| **Water Mass** | **Similarity Percentages (%)** | | |
| --- | --- | --- | --- |
|  | **Pacific Only** | **Indian Only** | **Combined** |
| **Bacteria** | | | |
| AABW | 29.4 | 22.4 | 21.9 |
| LCDW | 36.5 | 46.1 | 34.8 |
| UCDW | 32.9 | 32.8 | 29.5 |
| AAIW | 56.8 | 58.2 | 44.2 |
| AASW | 51.2 | 50.9 | 44.7 |
| SAMW | 51.9 | 57.6 | 38.3 |
| SASW | 55.6 | 56.1 | 33.3 |
| SICW | - | 71.9 | 71.9 |
| AAIW x UCDW | - | 46.6 | 46.6 |
| AAIW x SAMW | 42.0 | - | 42.0 |
| **Archaea** | | | |
| AABW | 53.7 | 37.5 | 38.7 |
| LCDW | 56.9 | 59.7 | 52.4 |
| UCDW | 51.1 | 48.4 | 44.9 |
| AAIW | 57.1 | 56.2 | 48.1 |
| AASW | 63.1 | 35.0 | 35.2 |
| SAMW | 58.3 | 46.8 | 36.7 |
| SASW | 62.6 | 48.1 | 36.0 |
| SICW | - | 66.9 | 66.9 |
| AAIW x UCDW | - | 56.6 | 56.6 |
| AAIW x SAMW | 47.4 | - | 47.4 |

**Table S7**: Pearson correlation of environmental variables considered within this study. ^+^Surface samples analyzed include all samples taken from depths < 20m from all stations.

|  | **Pressure** | **Latitude** | **Longitude** | **Temperature** | **Salinity** | **Oxygen** | **Phosphate** | **Silicate** | **Nitrite** | **NOx** | **Day-length** |
| --- | --- | --- | --- | --- | --- | --- | --- | --- | --- | --- | --- |
| **All Water Masses** | | | | | | | | | | |  |
| **Pressure** | - |  |  |  |  |  |  |  |  |  |  |
| **Latitude** | 0.033 |  |  |  |  |  |  |  |  |  |  |
| **Longitude** | -0.072 | 0.008 |  |  |  |  |  |  |  |  |  |
| **Temperature** | -0.171 | -0.751 | -0.203 |  |  |  |  |  |  |  |  |
| **Salinity** | 0.613 | -0.305 | -0.282 | 0.300 |  |  |  |  |  |  |  |
| **Oxygen** | -0.596 | 0.292 | 0.255 | -0.220 | -0.876 |  |  |  |  |  |  |
| **Phosphate** | -0.504 | -0.126 | 0.112 | 0.261 | -0.442 | 0.639 |  |  |  |  |  |
| **Silicate** | 0.811 | 0.399 | 0.048 | -0.500 | 0.520 | -0.568 | -0.657 |  |  |  |  |
| **Nitrite** | -0.557 | 0.021 | 0.051 | 0.133 | -0.593 | 0.734 | 0.595 | -0.665 |  |  |  |
| **NOx** | -0.529 | -0.172 | 0.037 | 0.295 | -0.407 | 0.617 | 0.923 | -0.688 | 0.582 |  |  |
| **Day-length** | 0.043 | -0.191 | -0.943 | 0.338 | 0.360 | -0.308 | -0.147 | -0.117 | -0.031 | -0.059 | - |
| **Surface Water^+^ Only** | | | | | | | | | | |  |
| **Pressure** | - |  |  |  |  |  |  |  |  |  |  |
| **Latitude** | -0.606 |  |  |  |  |  |  |  |  |  |  |
| **Longitude** | -0.149 | 0.185 |  |  |  |  |  |  |  |  |  |
| **Temperature** | 0.333 | -0.813 | -0.176 |  |  |  |  |  |  |  |  |
| **Salinity** | 0.275 | -0.566 | -0.575 | 0.666 |  |  |  |  |  |  |  |
| **Oxygen** | -0.285 | 0.746 | 0.594 | -0.706 | -0.753 |  |  |  |  |  |  |
| **Phosphate** | -0.069 | -0.312 | -0.028 | 0.304 | 0.036 | -0.261 |  |  |  |  |  |
| **Silicate** | -0.492 | 0.787 | 0.217 | -0.652 | -0.427 | 0.652 | -0.507 |  |  |  |  |
| **Nitrite** | 0.375 | -0.496 | -0.324 | 0.475 | 0.445 | -0.510 | 0.203 | -0.654 |  |  |  |
| **NOx** | 0.007 | -0.441 | -0.183 | 0.357 | 0.141 | -0.439 | 0.935 | -0.594 | 0.262 |  |  |
| **Day-length** | 0.352 | -0.401 | -0.917 | 0.376 | 0.717 | -0.699 | -0.046 | -0.422 | 0.514 | 0.125 | - |
| **AABW Only** | | | | | | | | | | |  |
| **Pressure** | - |  |  |  |  |  |  |  |  |  |  |
| **Latitude** | -0.289 |  |  |  |  |  |  |  |  |  |  |
| **Longitude** | -0.317 | 0.100 |  |  |  |  |  |  |  |  |  |
| **Temperature** | 0.034 | -0.715 | -0.550 |  |  |  |  |  |  |  |  |
| **Salinity** | -0.146 | -0.701 | -0.408 | 0.959 |  |  |  |  |  |  |  |
| **Oxygen** | 0.047 | 0.526 | 0.675 | -0.950 | -0.901 |  |  |  |  |  |  |
| **Phosphate** | -0.213 | 0.170 | 0.275 | -0.175 | -0.197 | 0.184 |  |  |  |  |  |
| **Silicate** | 0.325 | 0.476 | 0.141 | -0.694 | -0.725 | 0.560 | -0.028 |  |  |  |  |
| **Nitrite** | -0.003 | 0.424 | 0.360 | -0.528 | -0.509 | 0.510 | 0.495 | 0.300 |  |  |  |
| **NOx** | -0.346 | 0.127 | 0.169 | -0.059 | -0.105 | 0.046 | 0.715 | -0.138 | -0.059 |  |  |
| **Day-length** | 0.280 | -0.236 | -0.948 | 0.645 | 0.541 | -0.746 | -0.284 | -0.168 | -0.490 | -0.169 | - |

## Appendix S1: Supplementary methods

### Hydrological profiles defining Southern Ocean Water Masses and Fronts

Lower oxygenated zones (Pacific - 175.4-217.6 μmol L^-1^; Indian – 154.6-200 μmol L^-1^) defined the UCDW from LCDW, and salinity minima (33.8 – 34.3; PSS-78) distinguished AAIW from SAMW (Gordon, 1967). At the Pacific photic zone (within the mixed layer depth), sharp changes in temperature and salinity profiles at approximately 48, 55, 60 and 65°S indicate the locations of Subtropical, Subantarctic, Polar and southern Antarctic circumpolar current (sACC) fronts defining the different surface water zones (Figure 1b). Approximate locations of fronts mentioned above were observed within temperature profiles of the Indian sector at 41, 48, 51, 57°S, with the Southern Boundary at 64°S (Figure 1c).

Within the mixed layer (upper 250m) of the P15S transect, NOx (NO_3_^-^ + NO_2_^-^) concentrations increase from 4 to 33 μmol L^-1^ between 45 to 66°S. Minimum (20 μmol L^-1^) surface NOx concentrations within the Indian sector were observed between 48 to 52°S, peaking at 55-58°S (26 μmol L^-1^) before declining (17 μmol L^-1^) again towards the colder Antarctic waters. Nitrite decreased to levels nearing zero past the mixed layer, at all measured latitudes and sectors within the SO (Figure S1, S2), in alignment with commonly observed distributions (Lomas and Lipschultz, 2006).

### Sample collection devices and sensors

From the R/V Investigator, water was collected on a rosette water sampler mounted with the SBE911 conductivity, temperature and depth (CTD) sensors (Seabird Scientific, USA), SBE43 dissolved oxygen sensor (Seabird Scientific, USA), Aquatracka fluorometer (Chelsea Technologies, UK) *and Wetlabs C-Star™ transmissometer (Seabird Scientific, USA).* The third voyage was on the Aurora Australis icebreaker: Kerguelen Axis (K-Axis) voyage (11^th^ January-24^th^ February 2016) samples were collected using a rosette sampler mounted with SBE9plus CTD (Seabird Scientific, USA) with similar sensors to those outlined by Bestley et al. (2018).

### Nutrients Analysis

Silicate was analysed based on a modified version of the method by Armstrong et al. (1967), with minor changes to the coil sequence as recommended in Practical Guidelines for the Analysis of Seawater (Aminot et al., 2009) with detection limits of 0.2 µmol L^-1^. Phosphate was analysed based on the original Murphy and Riley (1962) method, using 10mm flow cells and a LED lamp with optimized antimony catalyst/phosphate ratio and reduction of silicate interferences by pH (detection limit 0.01 µmol L^-1^). Nitrate and Nitrite were measured using 10mm flow cells and a LED lamp according to the AA3 method G-172-96 rev15 based on Armstrong et al. (1967) and Grasshoff et al. (2009)(detection limits 0.015 µmol L^-1^). Unless elsewhere mentioned, NO_x_ is the sum of NO_3_^-^ and NO_2_^-^. Certified reference materials (CRMs) in seawater for each of silicate, phosphate, nitrate and nitrite (KANSO, Japan) were used to ensure accuracy. Reference CRMs were run 4 times following running of calibration standards, and accuracy was confirmed by cross checking batches of new standards against older batches and ensuring accuracy fell within 1% between batches. Acceptable levels of accuracy under the criteria of the GO-SHIP reference sections is specified as 1-3% of the full scale, depending on the nutrient analyzed (Hydes et al., 2010).

### Access to Hydrological, nutrients and other metadata

Physical, biogeochemical, nutrient and metadata discussed here (temperature, salinity, dissolved oxygen, NO_x_, NO_2_^−^, PO_4_^3−^ and Si) are accessible through the CLIVAR and Carbon Hydrographic Data Office (CCHDO) webpage (<https://cchdo.ucsd.edu/cruise/096U20160426>) for the P15S voyage, the Commonwealth Scientific and Industrial Research Organisation (CSIRO) Oceans and Atmosphere Data Centre webpage (<https://www.cmar.csiro.au/data/trawler/index.cfm>) for the HEOBI voyage, and the Australian Antarctic Data Centre (<https://data.aad.gov.au/kaxis>) for the K-Axis voyage.

### DNEasy Sterivex Extraction Protocol as Modified by Appleyard et al. (2013)

**Materials:**

Lysis buffer Stock Solutions

200mM NaH2PO42H2O (monobasic) (MW 156, 156/1L = 1M, 6.24/200 mL = 200 mM in 200 mL solution)

200mM Na2HPO4 (dibasic) (MW 142, 142g/1L =1M, 5.68g/200mL = 200mM in 200 mL solution)

To make up 200mL Lysis buffer:

39mL 200mM NaH2PO4 (monobasic)

61mL 200mM Na2HPO4 (dibasic)

17.54g NaCl

2g CTAB

4g PVP K30

Add distilled, deionized water to make up to 200ml

Adjust to pH 7.0 (using NaOH – try couple of mL of 10M NaOH)

From FastDNA™ Spin Kit for Soil (MP Biomedicals):

MT buffer

From DNEasy PowerWater Sterivex Kit (Qiagen):

Columns and sample recovery tubes, 3ml and 20ml syringes, 5ml tubes

Solution MR (contains 3M guanidinium thiocyanate high concentration salt solution) (warmed

to 65°C before use)

Ethanol

Solution PW (contains ethanol)

Inlet and outlet caps for Sterivex filters

Lysozyme

Proteinase K – 20 mg/ml

Phenol:Chloroform:Isoamyl (25:24:1) (PCI)

Chloroform:Isoamyl (24:1) (CI)

TE buffer

**Protocol:**

1. Weigh 125 mg lysozyme into 50 mL falcon tube and add 25 mL Lysis Buffer to dissolve (lysozyme final concentration 5 mg/ml).
2. Remove filters from -80°C, remove inlet cap and using a pipette add 1.875 ml Lysis buffer (containing 5mg/ml final concentration of lysozyme) and 0.125 ml MT buffer.
3. Recap the Sterivex filter and attach filter (with inlet end facing out) to the horizontal vortexer, speed 5-7 for 60 min (turning the filter a couple of times during the hour)
4. Using 3 ml syringe, draw back plunger and attach to inlet end of filter until pressure builds up – release plunger and buffer in filter should flow into syringe. Divide approximately 2 ml of buffer evenly into 2 x 2.0 ml tubes (do not use the 2 mL collection tubes that come in the PowerWater kit – they don’t tolerate PCI) (may need to use syringe several times to get all buffer out of filter, should be about 0.800-1.00 ml per tube)
5. In fume hood, add 900 µl PCI to each tube, invert several times, spin down 13000 rpm for 10 mins at room temperature.
6. Combine the aqueous phases from both tubes into one 2.0 ml tube (which will be between 1.2 – 1.5 ml), add 20 µl Proteinase K, onto heat block for 2 hours at 60°C
7. In fume hood, add 500 µl CI, spin down 13000 rpm for 10 mins at room temperature – put aqueous phase into new tube
8. In fume hood, add a further 500 µl CI, spin down 13000 rpm for 5 mins at room temperature – put aqueous phase into new tube
9. After 2^nd^ spin, take out 1 ml of aqueous phase, add to 5 ml tube
10. Add 3 ml of warmed MR buffer (65°C), mix by inversion
11. Attach column to barrel of 20 ml syringe and attach to vacuum manifold
12. Pour contents of 5 ml tube into barrel while still warm
13. Using vacuum, pull contents through the column
14. While keeping column attached to the manifold, remove barrel and add 800 µl ethanol to column. Using vacuum, pull contents through the column
15. Add 800 µl Solution PW to column. Using vacuum, pull contents through the column, then keep on vacuum for 2 mins
16. Turn vacuum off, put column into new 2.0 ml tube and let air dry on bench for 10 mins
17. Add 80 µl 0.1 x TE buffer to column, incubate at 37°C for 45 min
18. Spin down column and tube at 13000 rpm for 2 mins at room temperature to elute DNA

### Sequence Data Analyses

The quality of the paired reads were evaluated using FastQC (Andrews, 2014), then trimmed and merged using FLASH (Magoc and Salzberg, 2011) after the methods described in Bissett et al. (2016). Quality filtered sequences are mapped to biologically correct, chimera-free zOTUs using USEARCH 64 bit v10.0.240 (Edgar, 2010) and a sample by read abundance matrix is generated. Bacterial and Archaeal zOTUs were taxonomically identified with the SILVA database (v132) (Quast et al., 2013) and Genome Taxonomy Database (GTDB, release 89.0) (Parks et al., 2018), respectively using DADA2’s implementation of the Wang classifier with a 60% Bayesian probability cut-off (Wang et al., 2007; Callahan et al., 2016). Taxonomic identification of the archaeal zOTUs with GTDB facilitated an improved archaeal taxonomic classification by enabling the classification of many archaeal zOTUs to be resolved at the species level. Sequences that were unidentified or incorrectly identified at the kingdom or phylum level, as well as chloroplast and mitochondrial sequences were removed from the abundance matrix prior to further analysis.

### Distance-based linear models (DistLM)

DistLM was used to model and assess the effects of changes in measured environmental and geographical variables on community dissimilarity. The ‘BEST’ selection procedure, where all possible combinations of predictor variables are examined, was used with adjusted R^2^ as the selection criteria with 9999 permutations of model residuals under a reduced model. The model was created for all samples and subsets of surface and AABW samples. Where two environmental predictors were highly correlated (Pearson’s r > |0.85|) (Table S7), the predictor observed to contribute less to the proportion of community variation explained during preliminary model optimization were removed from the final models (i.e. phosphate and salinity from models considering all water masses and sectors; phosphate and salinity from models considering surface only samples; oxygen and nitrate/phosphate from models considering AABW only samples).

### Geographical distance calculation for distance-decay plots

To further test the effect of spatial distance on community similarity, plots of zOTU Bray Curtis similarity values versus geographical distance between the samples were generated. Actual geographical distances between sampling site pairs were calculated based on the formula d = acos(sin ϕ1 · sin ϕ2 + cos ϕ1 · cos ϕ2 · cos Δλ) · R, where ϕ1 is the latitude of the first site, ϕ2 is the latitude of the second site, Δλ is the longitudinal difference between the site pairs, and R is the Earth’s radius (6,371 km). The non-parametric Mantel test based on the Spearman correlation coefficient was applied and significance was assessed based on 1000 Monte Carlo permutations.

## Supplementary References

Aminot, A., Kérouel, R., and Coverly, S.C. (2009) Nutrients in seawater using segmented flow analysis. In *Practical guidelines for the analysis of seawater*, pp. 143-178.

Andrews, S. (2014) FastQC: a quality control tool for high throughput sequence data. In.

Appleyard, S., Abell, G., and Watson, R. (2013) Tackling microbial related issues in cultured shellfish via integrated molecular and water chemistry approaches. In *Seafood CRC Final Report (2011/729)*. Deakin, Australia: Australian Seafood Cooperative Research Centre, p. 89.

Armstrong, F.A.J., Stearns, C.R., and Strickland, J.D.H. (1967) The measurement of upwelling and subsequent biological process by means of the Technicon Autoanalyzer® and associated equipment. *Deep Sea Research and Oceanographic Abstracts* **14**: 381-389.

Bestley, S., van Wijk, E., Rosenberg, M., Eriksen, R., Corney, S., Tattersall, K., and Rintoul, S. (2018) Ocean circulation and frontal structure near the southern Kerguelen Plateau: The physical context for the Kerguelen Axis ecosystem study. *Deep Sea Res II Top Stud Oceanogr* **174**.

Bissett, A., Fitzgerald, A., Meintjes, T., Mele, P.M., Reith, F., Dennis, P.G. et al. (2016) Introducing BASE: the Biomes of Australian Soil Environments soil microbial diversity database. *Gigascience* **5**: 21.

Callahan, B.J., McMurdie, P.J., Rosen, M.J., Han, A.W., Johnson, A.J., and Holmes, S.P. (2016) DADA2: High-resolution sample inference from Illumina amplicon data. *Nat Methods* **13**: 581-583.

Edgar, R.C. (2010) Search and clustering orders of magnitude faster than BLAST. *Bioinformatics* **26**: 2460-2461.

Gordon, A.L. (1967) Structure of Antarctic waters between 20°W and 170°W. In *Antarctic Map Folio Ser*. C., B.V. (ed): Am Geogr Soc, p. 10.

Grasshoff, K., Kremling, K., and Ehrhardt, M. (2009) *Methods of seawater analysis*. Weinheim, Germany: Wiley-VCH.

Hydes, D., Aoyama, M., Aminot, A., Bakker, K., Becker, S., Coverly, S. et al. (2010) Determination of dissolved nutrients (N, P, Si) in seawater with high precision and inter-comparability using gas-segmented continuous flow analysers. In: UNESCO/IOC.

Lomas, M.W., and Lipschultz, F. (2006) Forming the primary nitrite maximum: Nitrifiers or phytoplankton? *Limnology and Oceanography* **51**: 2453-2467.

Magoc, T., and Salzberg, S.L. (2011) FLASH: fast length adjustment of short reads to improve genome assemblies. *Bioinformatics* **27**: 2957-2963.

Murphy, J., and Riley, J.P. (1962) A modified single solution method for the determination of phosphate in natural waters. *Analytica Chimica Acta* **27**: 31-36.

Parks, D.H., Chuvochina, M., Waite, D.W., Rinke, C., Skarshewski, A., Chaumeil, P.A., and Hugenholtz, P. (2018) A standardized bacterial taxonomy based on genome phylogeny substantially revises the tree of life. *Nat Biotechnol* **36**: 996-1004.

Quast, C., Pruesse, E., Yilmaz, P., Gerken, J., Schweer, T., Yarza, P. et al. (2013) The SILVA ribosomal RNA gene database project: improved data processing and web-based tools. *Nucleic Acids Research* **41**: D590-D596.

Wang, Q., Garrity, G.M., Tiedje, J.M., and Cole, J.R. (2007) Naïve Bayesian Classifier for Rapid Assignment of rRNA Sequences into the New Bacterial Taxonomy. *Applied and Environmental Microbiology* **73**: 5261-5267.
