# Supplementary material for: Biogeography of Southern Ocean prokaryotes: a comparison of the Indian and Pacific sectors
Source: Environ Microbiol. 2022 Feb 16;24(5):2449–66. doi: 10.1111/1462-2920.15906 (PMC9303206; doi:10.1111/1462-2920.15906)

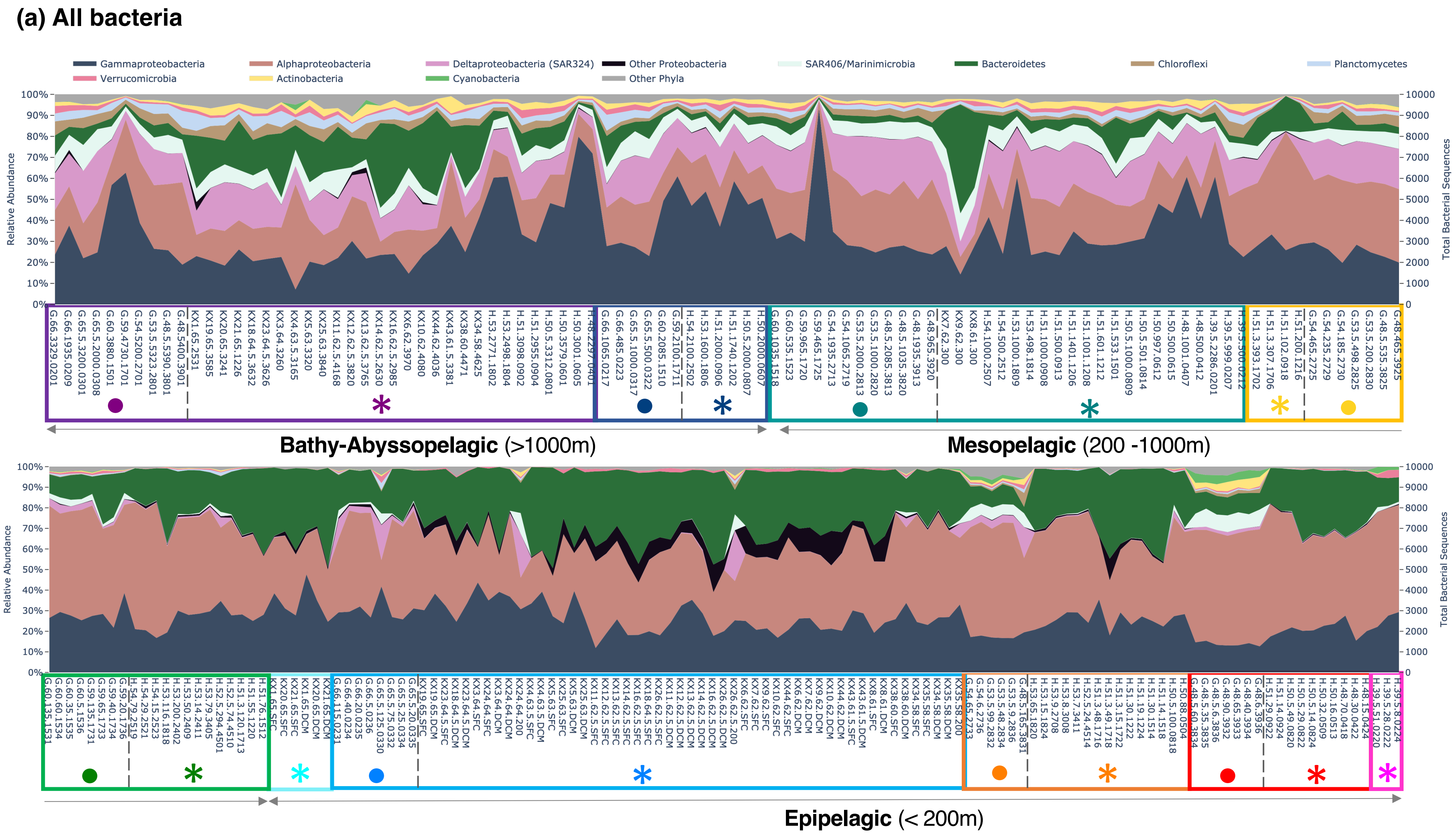

Fig. S6: (a) Full charts for relative abundance of major bacteria detected from the different water masses at phylum/class level, showing each of the 223 samples on the x-axis. Samples are arranged from high to low latitude in each water mass from the Pacific and Indian sector, as indicated by their respective sector/water mass symbols (see Fig. 1/Fig. 2 legends). Detailed charts of the main families within (b) Gammaproteobacteria, (c) Alphaproteobacteria and (d) Bacteroidetes is also shown. The full chart for archaeal relative abundance is shown in (e), and a detailed chart of the main *Nitrosopumilus* species/zOTUs is shown in (f).

(b) Gammaproteobacteria

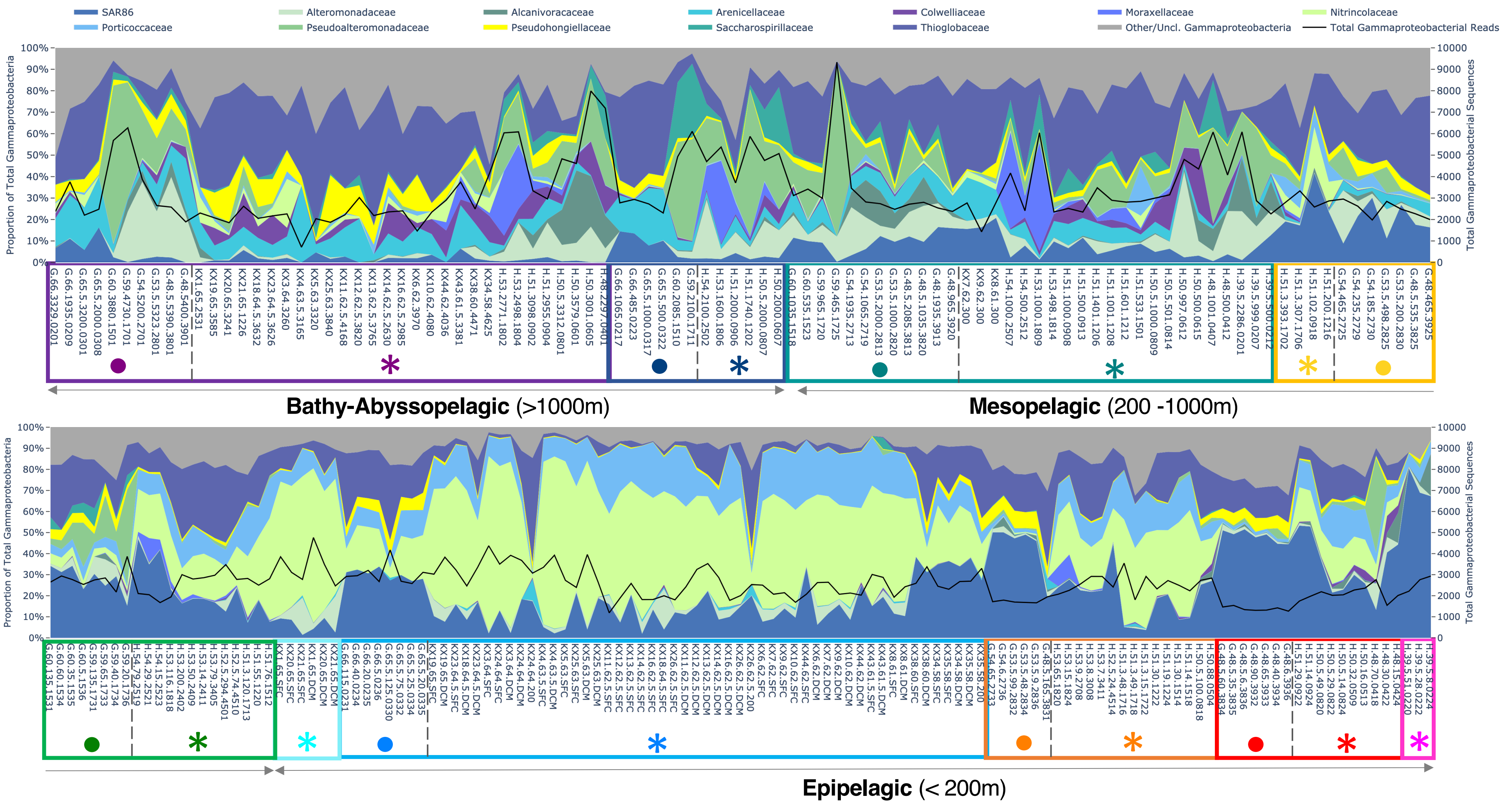

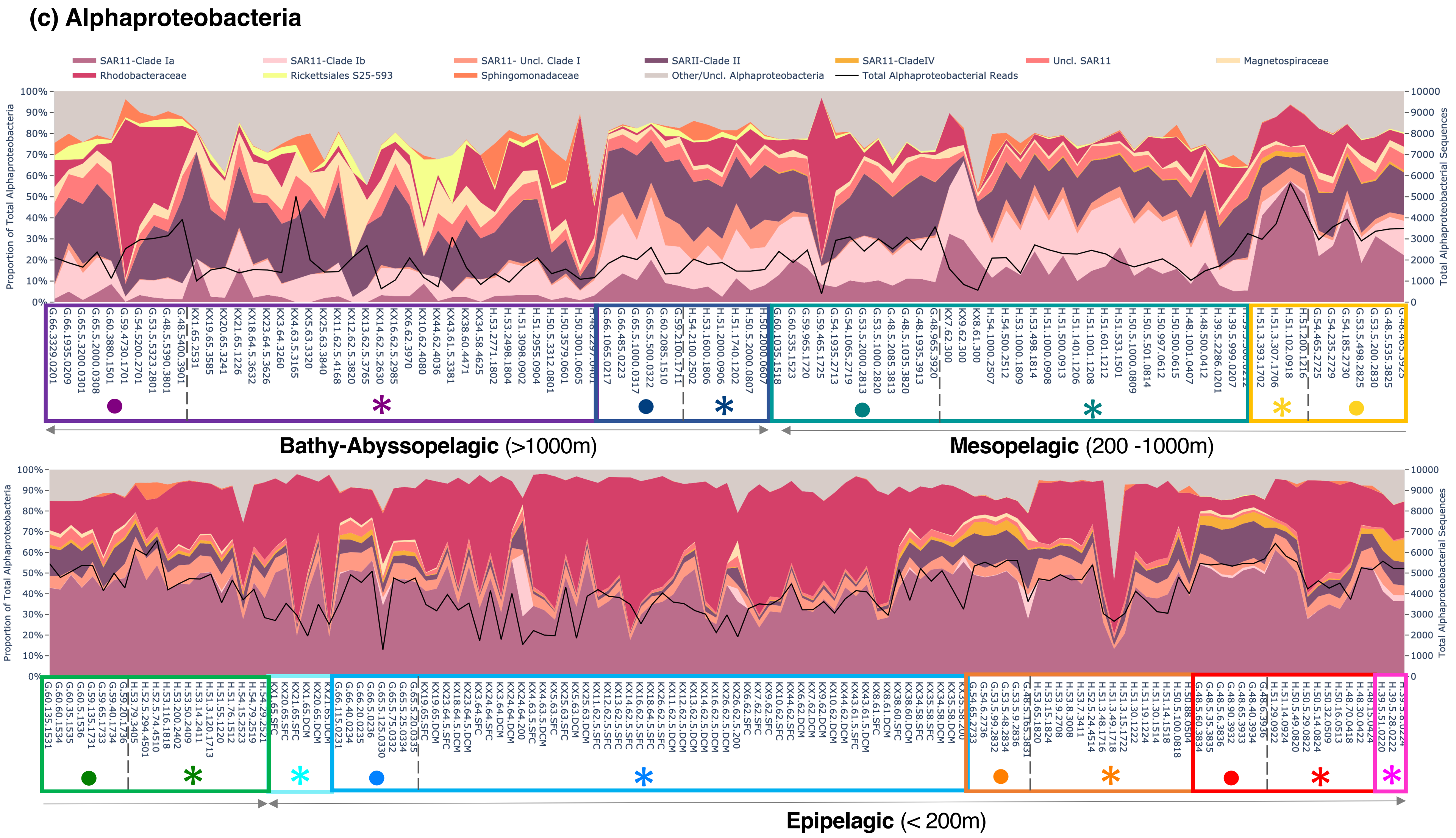
(c) Alphaproteobacteria
SAR11-Clade Ia
SAR11-Clade Ib
SAR11- Uncl. Clade I
SARII-Clade II
SAR11-CladeIV
Uncl. SAR11
Magnetospiraceae
Rhodobacteraceae
Rickettsiales S25-593
Sphingomonadaceae
Other/Uncl. Alphaproteobacteria
Total Alphaproteobacterial Reads
Proportion of Total Alphaproteobacteria
Total Alphaproteobacterial Sequences
Bathy-Abyssopelagic (>1000m)
Mesopelagic (200 -1000m)
Epipelagic (< 200m)

(d) Bacteroidetes

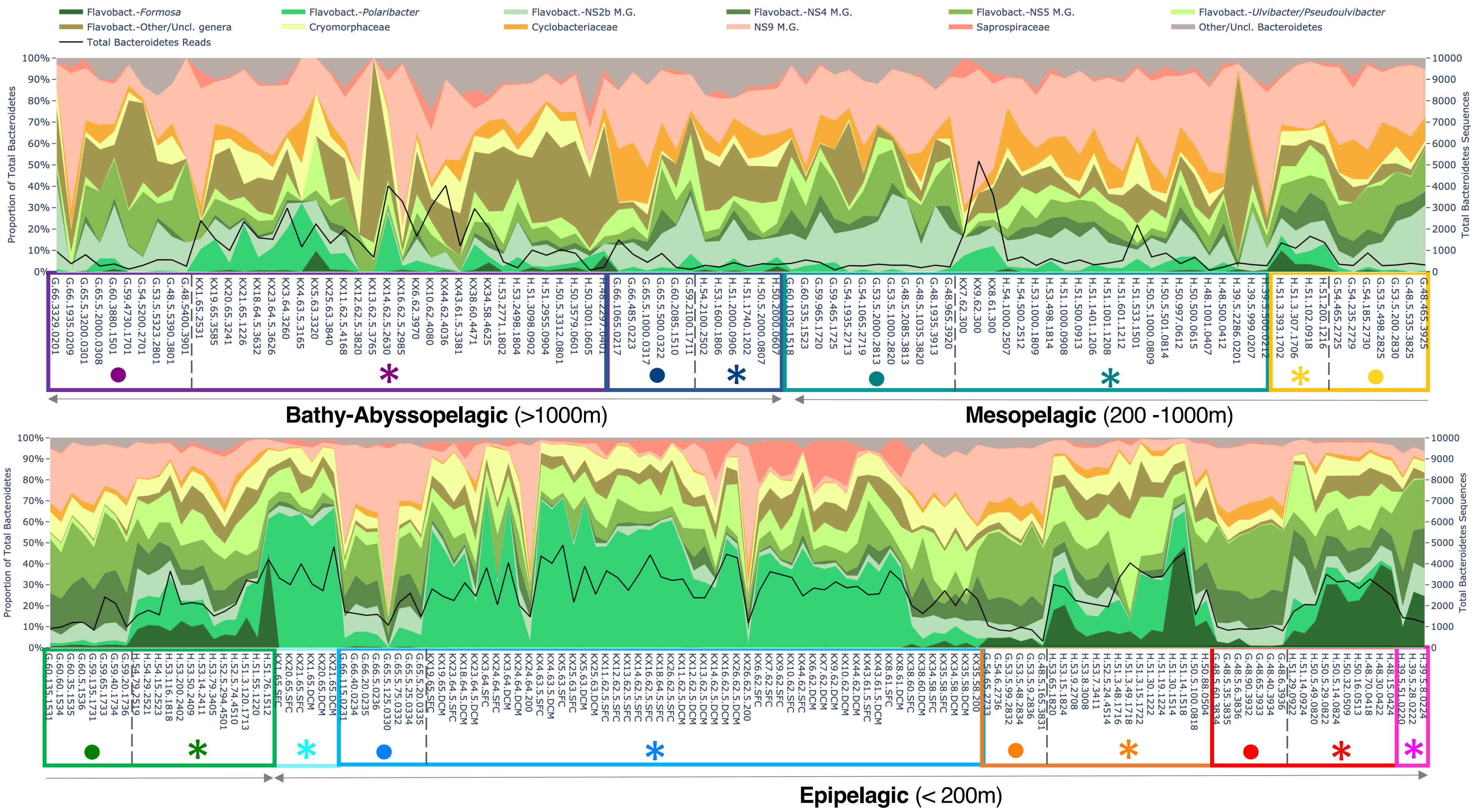

(e) All archaea

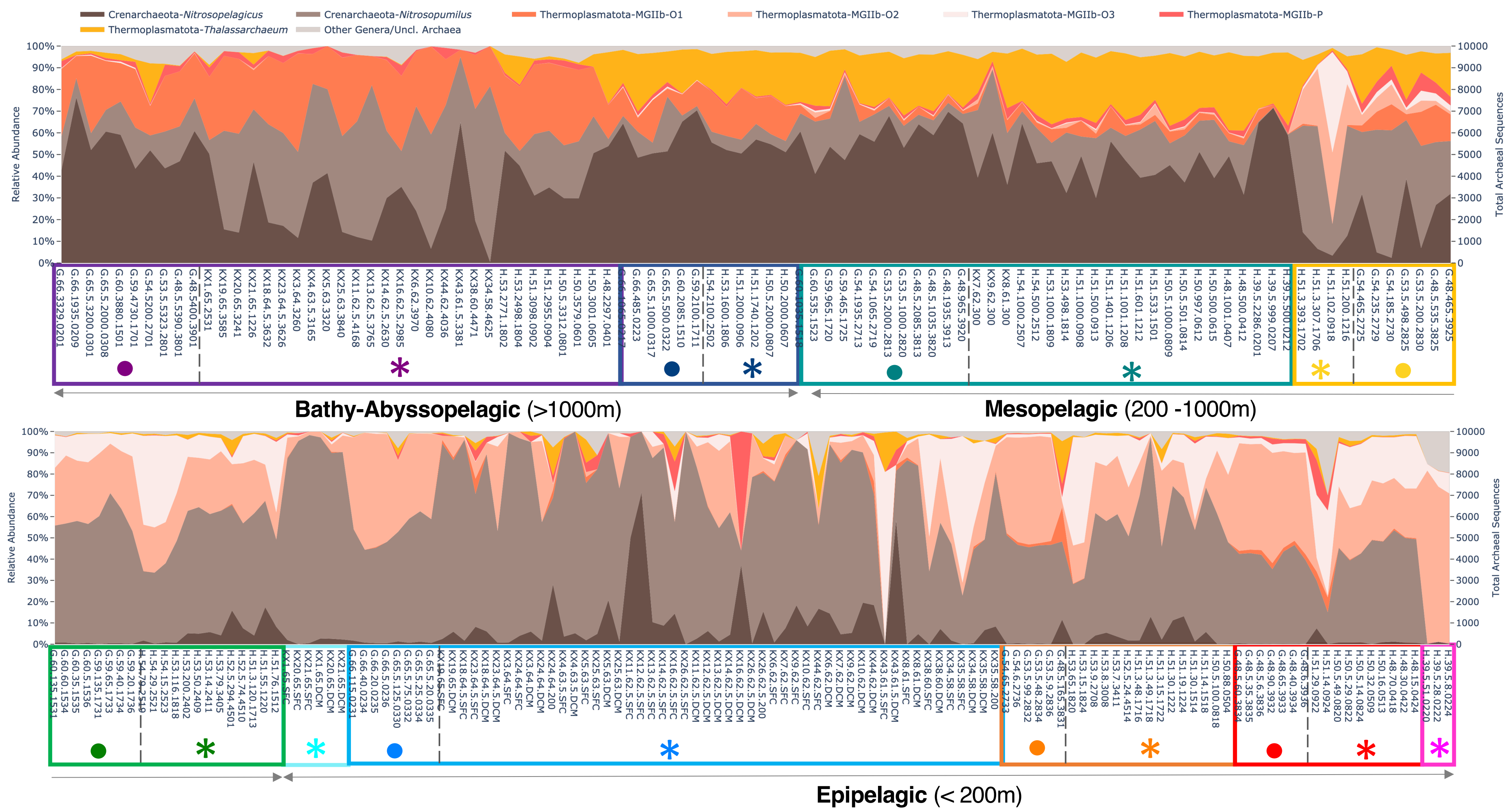

(f) *Nitrosopumilus*

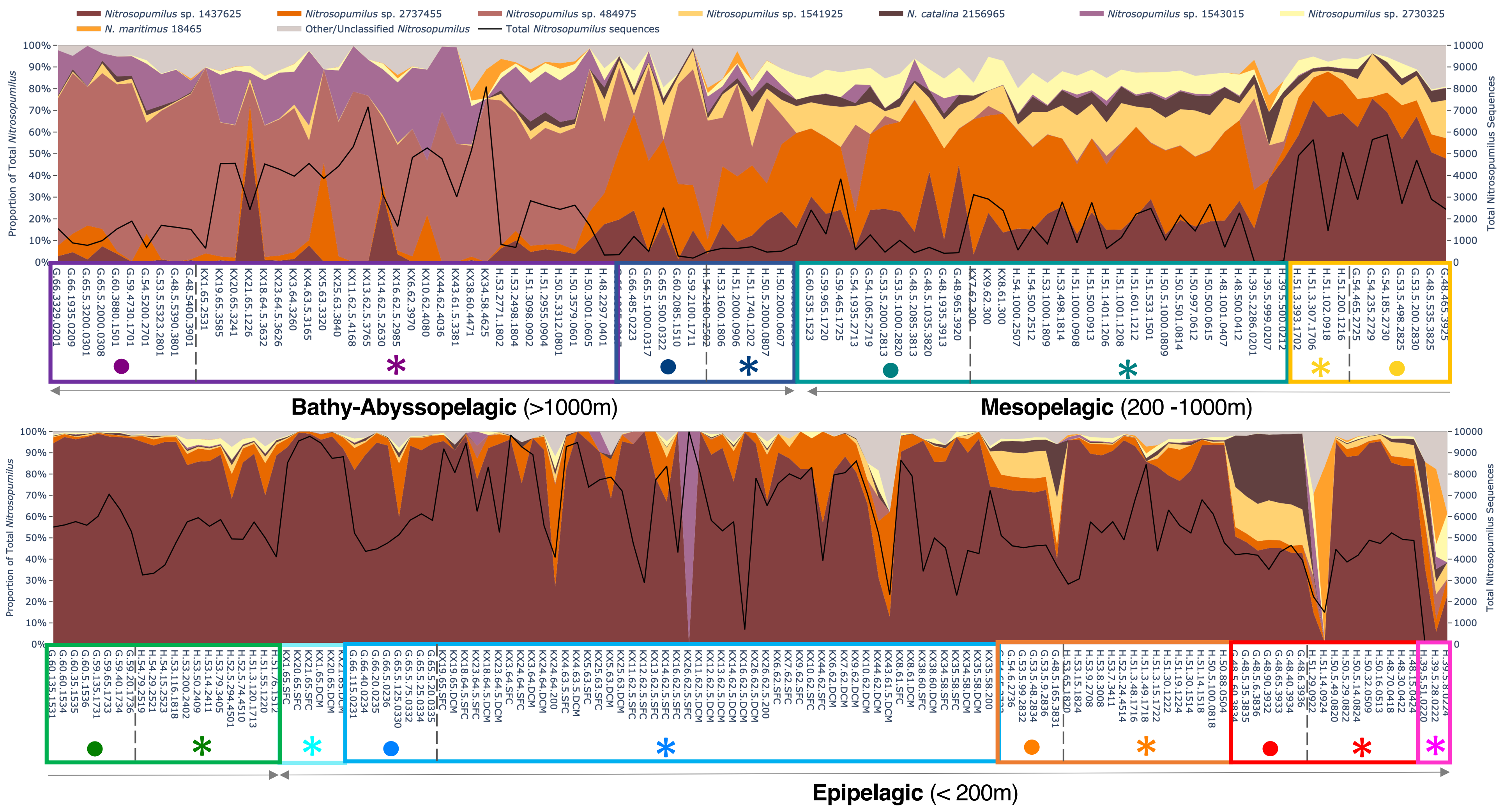

Supplement: Supplementary file 2 — Fig. S6. (a) Full charts for relative abundance of major bacteria detected from the different water masses at phylum/class level, showing each of the 223 samples on the x‐axis. Samples are arranged from high to low latitude in each water mass from the Pacific (P) followed by Indian (I) sector. Detailed charts of the main families within (b) Gammaproteobacteria, (c) Alphaproteobacteria and (d) Bacteroidetes is also shown. The full chart for archaeal relative abundance is shown in (e), and a detailed chart of the main Nitrosopumilus species/zOTUs is shown in (f). [file EMI-24-2449-s005.pdf]
